# Acute deletion of the central MR/GR steroid receptor correlates with changes in LTP, auditory neural gain, and GC-A cGMP signaling

**DOI:** 10.3389/fnmol.2023.1017761

**Published:** 2023-02-17

**Authors:** Dila Calis, Morgan Hess, Philine Marchetta, Wibke Singer, Julian Modro, Ellis Nelissen, Jos Prickaerts, Peter Sandner, Robert Lukowski, Peter Ruth, Marlies Knipper, Lukas Rüttiger

**Affiliations:** ^1^Department of Otolaryngology, Head and Neck Surgery, Tübingen Hearing Research Centre, Molecular Physiology of Hearing, University of Tübingen, Tübingen, Germany; ^2^Department of Psychiatry and Neuropsychology, School for Mental Health and Neuroscience (MHeNS), Maastricht University, Maastricht, Netherlands; ^3^Bayer Health Care Pharmaceuticals, Global Drug Discovery Pharma Research Centre Wuppertal, Wuppertal, Germany; ^4^Institute of Pharmacy, Pharmacology, Toxicology and Clinical Pharmacy, University of Tübingen, Tübingen, Germany

**Keywords:** glucocorticoid receptor, mineralocorticoid receptor, NO-GC, GC-A, cognition

## Abstract

The complex mechanism by which stress can affect sensory processes such as hearing is still poorly understood. In a previous study, the mineralocorticoid (MR) and/or glucocorticoid receptor (GR) were deleted in frontal brain regions but not cochlear regions using a CaMKIIα-based tamoxifen-inducible *Cre*^ERT2^/loxP approach. These mice exhibit either a diminished (MR^TMX^cKO) or disinhibited (GR^TMX^cKO) auditory nerve activity. In the present study, we observed that mice differentially were (MR^TMX^cKO) or were not (GR^TMX^cKO) able to compensate for altered auditory nerve activity in the central auditory pathway. As previous findings demonstrated a link between central auditory compensation and memory-dependent adaptation processes, we analyzed hippocampal paired-pulse facilitation (PPF) and long-term potentiation (LTP). To determine which molecular mechanisms may impact differences in synaptic plasticity, we analyzed Arc/Arg3.1, known to control AMPA receptor trafficking, as well as regulators of tissue perfusion and energy consumption (NO-GC and GC-A). We observed that the changes in PPF of MR^TMX^cKOs mirrored the changes in their auditory nerve activity, whereas changes in the LTP of MR^TMX^cKOs and GR^TMX^cKOs mirrored instead the changes in their central compensation capacity. Enhanced GR expression levels in MR^TMX^cKOs suggest that MRs typically suppress GR expression. We observed that hippocampal LTP, GC-A mRNA expression levels, and ABR wave IV/I ratio were all enhanced in animals with elevated GR (MR^TMX^cKOs) but were all lower or not mobilized in animals with impaired GR expression levels (GR^TMX^cKOs and MRGR^TMX^cKOs). This suggests that GC-A may link LTP and auditory neural gain through GR-dependent processes. In addition, enhanced NO-GC expression levels in MR, GR, and MRGR^TMX^cKOs suggest that both receptors suppress NO-GC; on the other hand, elevated Arc/Arg3.1 levels in MR^TMX^cKOs and MRGR^TMX^cKOs but not GR^TMX^cKOs suggest that MR suppresses Arc/Arg3.1 expression levels. Conclusively, MR through GR inhibition may define the threshold for hemodynamic responses for LTP and auditory neural gain associated with GC-A.

## Highlights

- Baseline MR suppresses GR levels.
- Enhanced GR suppresses presynaptic hippocampal excitability.
- Baseline MR and GR suppress hippocampal NO-GC.
- Baseline MR inhibits Arc/Arg3.1.
- Enhanced GR increases GC-A, LTP, and auditory neural gain.

## Introduction

Hearing dysfunction in response to age, acoustic trauma, or posttraumatic stress has been linked with different stress responses possibly influencing cognitive functions (Meltser and Canlon, 2011; Canlon et al., 2013; Basner et al., 2014; Jafari et al., 2019; Mazurek et al., 2019; Wang and Puel, 2020; Nadhimi and Llano, 2021). During stress, the naturally occurring glucocorticoid hormones (corticosterone in rodents and cortisol in humans) activate two different receptors. Besides aldosterone, the mineralocorticoid receptor (MR) shows high affinity to glucocorticoids, while the glucocorticoid receptor (GR) gradually becomes occupied by stress-induced high glucocorticoid concentrations (see for a review de Kloet et al., 2018). Both MR and GR actions need to be in balance for maintenance of homeostasis and health (see for a review de Kloet et al., 2018). The expression of MR dominates in glutamatergic neurons of the hippocampus, while GR is more ubiquitously expressed in the CNS (Reul and de Kloet, 1985; Chao et al., 1989; de Kloet et al., 2005; McEwen et al., 2016). To better understand the thus far elusive relationship between balanced stress receptor activity, hearing, and cognition, we previously employed a Cre/loxP-based approach in which a tamoxifen (TMX)-inducible Cre is expressed in the forebrain and hippocampus *via* the CaMKIIα promoter (Dragatsis and Zeitlin, 2000; Wang et al., 2013). The resulting MRGR^CaMKIIαCreERT2^ double knockout (MRGR^TMX^cKO) lacked MR and GR expression in frontal brain and in the hippocampal region, while cochlear MR and GR expression was unchanged (Marchetta et al., 2022). Remarkably, the examination of MR^CaMKIIαCreERT2^ single knockout (MR^TMX^cKO), GR^CaMKIIαCreERT2^ single knockout (GR^TMX^cKO), and MRGR^TMX^cKO mice unraveled unfavorable effects of central MR deletion and favorable effects of central GR deletion on peripheral auditory nerve processing (Marchetta et al., 2022), suggesting a top-down effect of central MR and GR activities. We reconsidered that the coordinated function of central MR and GR are predominantly predicted to influence the extinction (MR) and consolidation (GR) of long-term potentiation (LTP; see for a review de Kloet et al., 2018). Any adaptive central auditory responses following sound enrichment or acoustic trauma were found to be accompanied by increased hippocampal LTP levels (Matt et al., 2018; Likhtik and Johansen, 2019; Knipper et al., 2020; Marchetta et al., 2020b; Knipper et al., 2022; Manohar et al., 2022; Savitska et al., 2022; Zhang et al., 2022). The association between adaptive central auditory responses following sound enrichment or auditory trauma and LTP changes is suggested to be triggered through corticothalamic feedforward and feedback circuits (Antunes and Malmierca, 2021; Knipper et al., 2022). This encouraged us to investigate the influence of changed peripheral auditory processing in MR^TMX^cKO and GR^TMX^cKO (Marchetta et al., 2022) on central auditory responses of these animals in relation to short- and long-term hippocampal plasticity responses. Since the stress-induced drop in central adaptive auditory responses is restorable by inhibition of 3′,5′-cyclic guanosine monophosphate (cGMP) hydrolyzing phosphodiesterase 9A (Savitska et al., 2022), we additionally asked to what extent cGMP-producing nitric oxide (NO)-sensitive (NO-GC), encoded by the mammalian *Gucy1a1*, *Gucy1a2,* and *Gucy1b1* genes, and membrane-bound (GC-A) guanylyl cyclase, encoded by the mammalian *Npr1* gene, may correlate with changes in auditory or hippocampal circuits acutely induced by MR and/or GR deletion. This is of particular interest because cGMP plays an important role in AMPA and NMDA receptor signaling, facilitating synaptic plasticity and memory formation (Giesen et al., 2022), specifically including functions of NO-GC (Koesling et al., 2016; Nelissen et al., 2021, 2022) and possibly also GC-A (Kroker et al., 2012). As AMPA receptor surface diffusion during LTP was shown to be changed by GR activation (Groc et al., 2008), the mRNA of activity-regulated cytoskeletal protein (Arc, also known as Arg3.1) that controls AMPA receptor trafficking during LTP/long-term depression (LTD; Guzowski et al., 1999; Plath et al., 2006; Kuipers et al., 2016) was analyzed.

Based on the herein presented findings, we conclude that balanced basal MR and GR activities play a critical role in setting the threshold for presynaptic reactivities in the hippocampus. Basal MR expression may suppress GR expression levels, which enables higher levels of LTP. This dynamic setting of MR and GR activity appears to keep the threshold for presynaptic excitability (PPF through GR), the NO-GC level (through MR and GR), and neuronal Arc/Arg3.1 level (through MR) low. The elevation of GR levels may lead to elevated hippocampal GC-A and Arc/Arg3.1, elevated LTP, and elevated central auditory neural gain. We conclusively suggest that GR-induced changes in GC-A activity are involved in auditory neural gain, and thus may provide a means for altering corticofugal top-down feedback.

## Materials and methods

### Animals

Animal care, use, and experimental protocols correspond to national and institutional guidelines and were reviewed and approved by University of Tübingen, Veterinary Care Unit, and the Animal Care and Ethics Committee of the regional board of the Federal State Government of Baden-Württemberg, Germany. All experiments were performed according to the European Union Directive 2010/63/EU for the protection of animals used for experimental and other scientific purposes. In-house bred mice were kept in a specified pathogen free facility at 25°C on a 12/12 h light/dark cycle with average noise levels of around 50–60 dB SPL. The weight of the animals was recorded on each experimental day.

In the present study, three TMX-inducible conditional knockout mouse lines were studied, in which MR and/or GR were deleted mainly in the forebrain. MRGR^TMX^cKO, MR^TMX^cKO, GR^TMX^cKO and corresponding control animals were generated as previously described (Marchetta et al., 2022). In brief, homozygous floxed MR, GR (Berger et al., 2006; Erdmann et al., 2007), or MRGR lines, in which exon 3 of *Mr* and/or *Gr* is flanked by *loxP* sites, were crossed with a CaMKIIα CreERT2 line (kindly provided by Prof. Günther Schütz). After confirmation of normal hearing function, all mice received an intraperitoneal injection of 1 mg TMX in 100 μL TMX-solution (Sigma-Aldrich, T-5648, Munich) twice a day for five consecutive days at the lowest age of approximately eight weeks. 50 mg TMX were dissolved in 500 μL Ethanol abs. (Merck, Darmstadt) and 4.5 mL sunflower oil (Sigma-Aldrich, S-5007). After the last injection, animals were allowed to recover in their home cages for four weeks before experiments started. For the respective transgenic mouse line, homozygous floxed Cre-negative littermates, which also received TMX injections, were used as controls. For all lines, mice of both sexes ranging between 1.8 months (beginning of the experiment) and 7.9 months (end of the experiment) were used. The genetic status of all mouse lines was confirmed by genotyping using gene-specific PCR protocols.

### Hearing measurements

Mice were anesthetized with an intraperitoneal injection of a mixture of Fentanyl (0.05 mg/kg bodyweight (BW), Fentanyl-Hameln, Hameln Pharma plus, Hameln, Germany), Midazolam (5.0 mg/kg BW, Midazolam-hameln®; Hameln Pharma plus), Medetomidin (0.5 mg/kg BW, Sedator®; Albrecht, Aulendorf, Germany) and atropine sulfate (0.2 mg/kg BW, B. Braun, Melsungen, Germany) diluted with water ad. inj. (Ampuwa, Fresenius KABI, Bad Homburg, Germany) to an injection volume of 10 mL/kg BW. Additional doses of anesthetics were administered if needed. The anesthesia was antagonized after the measurements by a subcutaneously administered mixture of Naloxon (1.2 mg/kg BW, Naloxon-hameln®; Hameln Pharma plus), Flumazenil (0.55 mg/kg BW, Flumazenil®; Fresenius KABI), and Atipazemol (2.5 mg/kg BW, Antisedan®; VETOQUINOL GmbH, Ravensburg, Germany) diluted with water ad. inj. (Ampuwa) to an injection volume of 10 mL/kg BW.

The anesthetized mice lay on a pre-warmed resting pad (37°C) in a soundproof chamber (IAC 400-A, Industrial Acoustics Company GmbH, Niederkrüchten, Germany). Auditory brainstem responses (ABRs) in anesthetized mice were evoked by short-duration sound stimuli with the same stimulus parameters for all groups. The ABRs represent the summed activity of neurons in distinct anatomical structures along the ascending auditory pathway recorded from subcutaneous cranial electrodes. A microphone (Bruel & Kjaer 4191, Naerum, Denmark) was used to calibrate and record the acoustic stimuli. ABR thresholds were elicited with click stimuli (100 μs duration with an FFT mean of 5.4 kHz). The stimulus level was increased stepwise from 10 to 100 dB SPL in 5 dB steps. Stimuli were generated with an I-O-card (PCI-6052E, PCI-6251, or PCIe-6259, National Instruments, Austin, Texas, United States) in an IBM compatible computer. The SPL of the stimuli was modulated by custom-made amplifier and attenuator systems (Wulf Elektronik, Frankfurt, Germany). The measured signals were band-pass filtered from 200 Hz to 5 kHz (F1, 6-pole Butterworth hardware Filter, Wulf Elektronik) and amplified by 100,000. The analog/digital (A/D) rate was 20 kHz. Each stimulus had a recording interval of 16 ms and was directly repeated and averaged up to 512 times.

### Field excitatory postsynaptic potential recordings in hippocampal slices

Extracellular field excitatory postsynaptic potential (fEPSP) recordings were performed according to standard methods as previously described (Matt et al., 2011; Chenaux et al., 2016). In brief, 400 μm thick hippocampal brain slices were coronally sectioned on a vibratome (Leica VT 1000S, Wetzlar, Germany) in ice-cold dissection buffer (mM): 127 NaCl, 1.9 KCl, 1.2 KH2PO4, 26 NaHCO3, 10 D-glucose, 2 MgSO4, and 1.1 CaCl2, constantly saturated with 5% CO2 and 95% O2 (pH 7.4). Slices were incubated in carbogenated artificial cerebrospinal fluid (in mM: 127 NaCl, 1.9 KCl, 1.2 KH2PO4, 26 NaHCO3, 10 D-glucose, 1 MgSO4, 2.2 CaCl2; pH 7.4) for 1 h at 30°C and afterwards stored at room temperature. Recordings were performed in a submerged-type recording chamber (Warner Instruments, Holliston, MA, United States). Stimulation (TM53CCINS, WPI, Sarasota, FL, United States) and recording (artificial cerebrospinal fluid-filled glass pipettes, 2–3 MΩ) electrodes were positioned in the stratum radiatum to record Schaffer collateral fEPSPs. Signals were amplified with an Axopatch 200B (Molecular Devices, San Jose, CA, United States), digitized at 5 kHz with an ITC-16 (HEKA, Reutlingen, Germany) and recorded using WinWCP from the Strathclyde Electrophysiology Suite. Stimuli (100 μs) were delivered through the stimulus isolator (WPI). For each individual slice the strength of the stimulation (typically between 30 and125 μA) was chosen to evoke 40%–60% of the maximal response, defined by initial fEPSP slope. Only slices that showed stable fiber volley (FV) and fEPSP were used for further recording. The same stimulus intensity was applied during baseline recording (0.067 Hz, 20–30 min) and during induction of long-term potentiation (LTP) using 100 stimuli during 1 s (100 Hz). The baseline was determined by averaging fEPSP initial slopes from the period before the tetanic stimulation (at least 15 min of stable recording). The level of LTP was determined by averaging fEPSP slopes from the period between 50 and 60 min after the high-frequency stimulation (HFS). Before the tetanic stimulation, each slice was used to record paired-pulse facilitation [PPF, 10–20–50–100–200–500 ms interstimulus interval (ISI) at the same stimulation strength as LTP recordings]. The paired-pulse ratio of EPSP2/EPSP1 slope and amplitude at each ISI were defined per slice and mean values per group were plotted. EPSP1 was calculated as an average of EPSP1s from all ISIs for each single slice.

Four traces were averaged for each single data point analyzed.

### Riboprobe synthesis

To amplify Arc/Arg3.1, we used the following primers: for: 5′-CGA AGT GTC CAA GCA GGT G-3′; and rev: 5′-TGA TGG CAT AGG GGC TAA CA-3′. To amplify NO-GC, we used the following primers: for: 5′-ATC CTC TTC AGC GGC ATT GTG-3′ and rev: 5′-TGC ATT GGT TCC TTC TTG CCC-3′. To amplify GC-A, we used the following primers: for: 5′-TGT GAA ACG TGT GAA CCG GA-3′ and rev: 5′-AGG CGG ATC GTT GAA AGG G-3′. To amplify GR, we used the following primers: for: 5′-TCC CCC TGG TAG AGA CGA AG-3′ and rev: 5′-GGC TGG TCG ACC TAT TGA GG-3′. To amplify MR, we used the following primers: for: 5′-GAG ATG AGG CTT CTG GGT GT-3′ and rev: 5′-CAG GAT CAT GGA CGG GGA TG-3′. These fragments were cloned into the pCR II Topo vector (Invitrogen, Karlsruhe, Germany) and their nucleotide sequences were verified by an automated sequencer. Plasmids were isolated using QIAprep Spin Miniprep Kit from Qiagen (Hilden, Germany). Complementary strands for sense and antisense riboprobes were transcribed from either Sp6 or T7 RNA polymerases and labeled using rNTP mix containing digoxigenin labeled uridine triphosphates. All restriction enzymes, RNA polymerases and digoxigenin-labeled rNTP were purchased from Roche Diagnostics (Mannheim, Germany).

### Co-localization of mRNA and protein in brain sections

A separate subset of mice from those used for *in vitro* electrophysiology measurements was deeply anesthetized with CO2 and then sacrificed by decapitation. Brain tissue was prepared and sectioned with a vibratome at 60 μm, as previously described (Singer et al., 2013a, 2016). mRNA and protein were co-localized on free-floating brain sections as previously described (Singer et al., 2013a). In brief, following prehybridization for 1 h at 37°C, sections were incubated overnight with NO-GC, GC-A, Arc/Arg3.1, MR, or GR riboprobes at 56°C, incubated with anti-digoxigenin antibody conjugated to alkaline phosphatase (anti-Dig-AP, Roche, 11093274910), and developed as previously described (Singer et al., 2013a). For protein detection, streptavidin–biotin was blocked according to the manufacturer’s instructions (Streptavidin–Biotin Blocking Kit, Vector Laboratories, Newark, CA, United States) after blocking endogenous peroxidase. Sections were incubated overnight at 4°C with the primary antibody against parvalbumin (Abcam, Berlin, Germany, anti-rabbit, 1:500, ab11427) as a marker for inhibitory neurons, followed by incubation with the secondary antibody (biotinylated goat anti-rabbit, Vector Laboratories, BA-1000) and chromogenic detection (AEC, 3-amino-9-ethylcarbazole, Vector Laboratories, SK-4200). For microscopy (BX61 microscope, Olympus, Hamburg, Germany) evaluation photographs of the hippocampus and auditory cortex were taken at a bregma position between −1.58 and −2.18 with a bright-field camera (DP 71, Olympus) for detection of mRNA and protein, without adjusting the picture frame or the plane of focus.

### Identification of GRE binding sites in NO-GC and GC-A upstream regions

To identify potential glucocorticoid-responsive elements (GRE) binding sites in NO-GC and GC-A upstream regions, Benchling was used to import and annotate the following genes: *Gucy1a1*, *Gucy1a2, Gucy1b1,* and *Npr1*. Subsequently, known sequences for GRE binding sites were aligned (see Supplementary Methods). These sequences were based on JASPAR and sequences previously identified by Meijsing et al. (2009), Polman et al. (2013), and van Weert et al. (2017).

## Quantification and statistical analysis

All statistical information and *n*-numbers can be found in the results section and in Supplementary Table S1. Data was tested for a normal distribution (the Shapiro–Wilk normality test, α = 0.05). Differences of the means were compared for statistical significance either by unpaired two-tailed Student’s *t*-test (parametric)/Mann–Whitney *U*-test (non-parametric), repeated measurement (RM) 2-way ANOVA, 2-way ANOVA (parametric) with α = 0.05 and correction for type 1 error using Sidak’s and Bonferroni’s multiple comparisons tests.

In figures, significance and a trend for significance is indicated by asterisks [(*) *p* < 0.08, * *p* < 0.05, ** *p* < 0.01, *** *p* < 0.001]. n.s. denotes non-significant results (*p* ≥ 0.08). The *p*-values of the 2-way ANOVAs refer to the main effect of the genotype.

### ABR analysis

For each individual ear, the peak input–output function for amplitude of the click-ABR measurements were averaged for intensities between 0 and 40 dB relative to threshold (re thr) and analyzed as previously described (Chumak et al., 2016).

Two peak classes were selected: (1) early peaks (at 1.2–1.8 ms, wave I), interpreted as the sum of the first stimulus-related action potential within the auditory nerve, and (2) delayed peaks (at 4.1–4.9 ms, wave IV), the response from the auditory midbrain.

Wave IV/I ratio was calculated by dividing the ABR wave IV amplitude by ABR wave I amplitude for individual animals at all intensities higher than 5 dB (re thr). For each individual ear, stimulus levels of 10–30 dB, 35–55 dB, and 60–80 dB re thr were averaged to yield three repeated measurements for fibers of different sensitivity and spontaneous rate [high-spontaneous rate (SR) low-threshold fibers, middle-SR, and low-SR high-threshold fibers; Bharadwaj et al., 2014]. Inter-peak latency growth functions were calculated by subtracting the ABR wave I latency from the ABR wave IV amplitudes for individual animals for increasing stimulus levels with reference to the ABR thresholds (from higher than 5 dB to a maximum of 80 dB re thr) and grouped into stimulus level ranges as described (10–30 dB, 35–55 dB, and 60–80 dB). For statistical analyses, single ears are used as sample size.

### fEPSP recordings in hippocampal slices

Data was analyzed and processed using Clampfit 10 (Molecular Devices) and Excel (Microsoft). The data presented per experimental group/condition contained (in addition to mean ± SEM) single dots showing the fEPSP slope values for each individual brain slice. The *n* indicates the number of slices and animals (slices/animals) used in the analysis. Recordings which did not show stable baseline or shifting ±2% from the average in the baseline recording were not included in the statistical analysis. For statistical analyses, single slices are used as sample size.

### Co-localization of mRNA and protein and immunohistochemistry in brain sections

Brain sections were quantified by integrating density values of color pixels for each single specimen using ImageJ software (NIH, Bethesda, MD; United States). Quantification of the mRNA from the double method staining was performed by artificially separating the image into three defined color “channels,” which were selected as the average signal from all groups of the background, the protein staining, and the mRNA staining. A detection threshold was then defined in the mRNA color “channel” which was consistent across all groups and genotypes. The integrated density was then calculated for each image and all images are averaged for one animal. The density values of all specimens stained within the same experiment were then normalized to the group mean (i.e., all hippocampal brain sections stained in the same experiment gave an average value of 1.0). This correction allowed for compensation of the high intertrial variation of staining intensity. All sections from one mouse were then averaged and entered the statistical evaluation as *n* = 1. When no quantifiable staining could be measured, the group was excluded from analysis.

## Results

### Acute deletion of MR in adult mice leads to elevated GR expression levels

**Figure 1:**
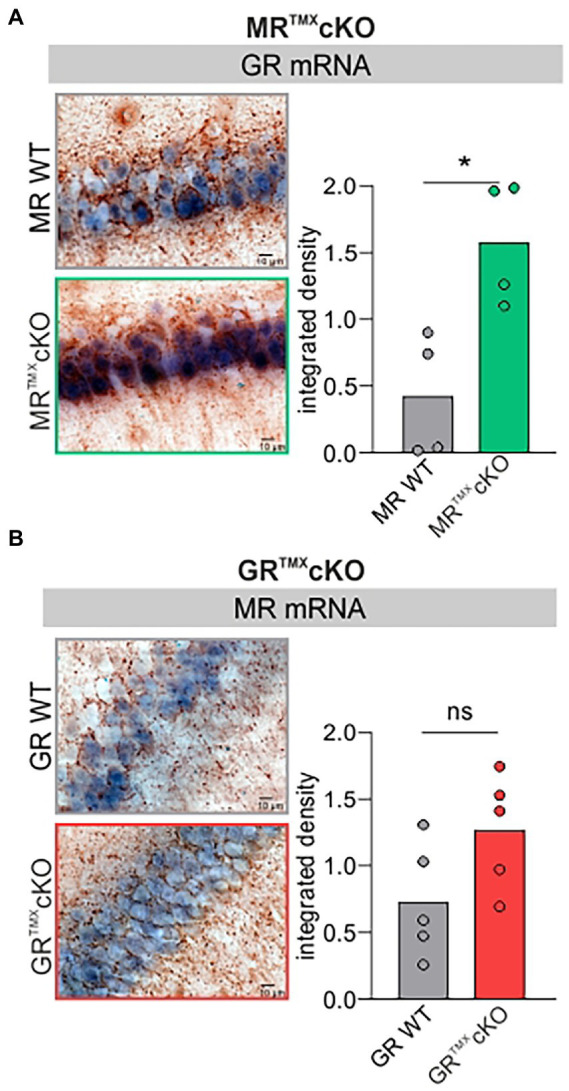
GR and MR mRNA expression level (blue) in the CA1 region of the hippocampus of MR and GR^TMX^cKO mice. **(A)** MR^TMX^cKO (green) mice showed significantly higher GR mRNA expression levels in comparison to their WT controls (gray). **(B)** GR^TMX^cKO (red) mice showed no difference in MR mRNA expression levels in comparison to their WT controls (gray). Mean ± SEM. ns = *p* > 0.08, * = *p* < 0.05.

Global and conditional MR deletion affected the expression profiles of GR in central neurons (Berger et al., 2006; Erdmann et al., 2007). We questioned if comparable effects are observed after TMX-induced acute deletion in MR and GR^TMX^cKOs. Brain sections of MR and GR^TMX^cKOs were exposed to GR- or MR-specific riboprobes. Data was evaluated quantitively as previously described (Singer et al., 2013a; Eckert et al., 2021). As exemplarily shown in Figure 1A, MR^TMX^cKOs had a significantly higher expression of GR mRNA in the hippocampus in comparison to their WT controls [Figure 1A, unpaired two-tailed student’s *t*-test, *t*(6) = 3.530, *p* = 0.0124, *n* = 4 mice each]. In contrast, conditional ablation of GR did not significantly affect the MR mRNA abundance in the hippocampus in comparison to WT controls [Figure 1B, unpaired two-tailed student’s *t*-test, *t*(8) = 1.993, *p* = 0.0814, *n* = 5 mice each]. The finding confirms that the differential GR expression which was observed in global and conditional MR/GR double- and single-KOs (Berger et al., 2006; Erdmann et al., 2007) is also observed in TMX-induced MR^TMX^cKOs. This proves the validity of the CaMKIIα-based TMX-inducible *Cre*^ERT2^/*loxP* approach for central deletion of MR and GR.

This also means that the MR^TMX^cKO phenotype should not only be interpreted with respect to the central MR deletion but also from the perspective of increased GR expression levels.

### Differential impact of central MR and/or GR deletion on central auditory neural gain, PPF, and LTP

**Figure 2:**
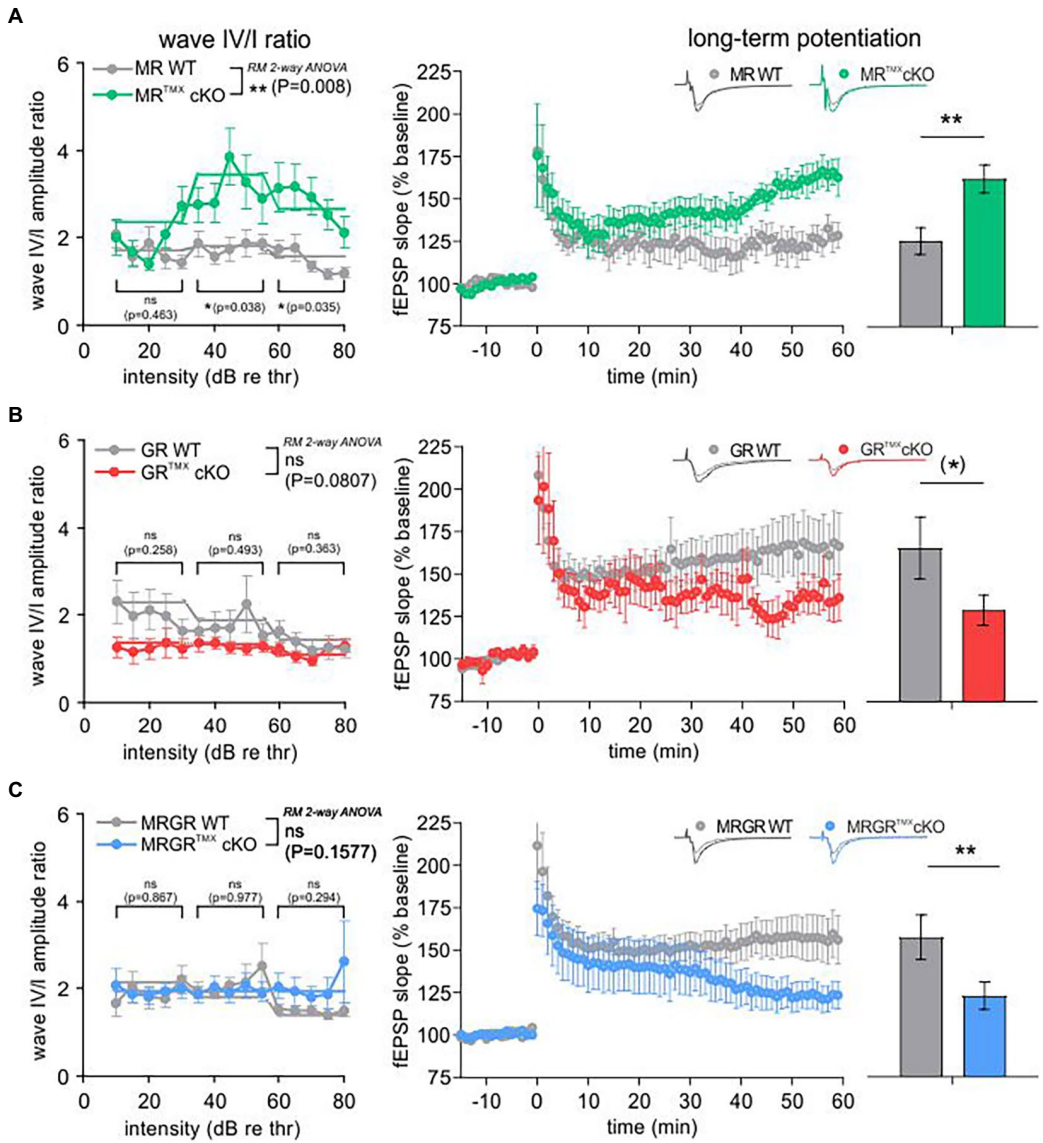
ABR wave IV/I amplitude ratio and LTP of MR^TMX^cKO, GR^TMX^cKO, and MRGR^TMX^cKO in comparison to their WT controls. **(A)** MR^TMX^cKOs (green) had a significantly higher wave IV/I ratio in comparison to their WT controls (gray). The MR^TMX^cKOs had significantly higher LTP in comparison to their WT controls. **(B)** GR^TMX^cKOs (red) had a slightly, non-significantly lower wave IV/I ratio compared to their WT controls (gray). GR^TMX^cKOs had a trend toward lower LTP in comparison to their WT controls. **(C)** MRGR^TMX^cKOs (blue) did not show a significant difference of wave IV/I ratio in comparison to their WT controls (gray). Contrary to this, MRGR^TMX^cKOs had significantly lower LTP in comparison to their WT controls. Mean ± SEM. ns = *p* > 0.08, (*) = *p* < 0.08, * = *p* < 0.05, ** = *p* < 0.01.

We next analyzed the central auditory responses (ABR wave IV) relative to the altered cochlear auditory processing (ABR wave I) of MR^TMX^cKOs, GR^TMX^cKOs, and MRGR^TMX^cKOs mice (Figure 2, left). Raw ABR wave amplitude and latency values were consistent with previously-reported data for all genotypes (data not shown; Marchetta et al., 2022). ABR wave IV/I ratio (input/output function) analysis for stimulus ranges corresponding to auditory fibers of different sensitivity and response range [10–30 dB, 35–55 dB, and 60–80 dB re thr for high-spontaneous rate (SR), middle-SR, and low-SR auditory fibers, respectively (Bharadwaj et al., 2014)] revealed that the reduced auditory nerve response of MR^TMX^cKOs could be centrally compensated through neural gain [higher ABR wave IV/I ratio; Figure 2A, left, repeated measurement (RM) 2-way ANOVA, *F*(1, 49) = 7.652, *p* = 0.008, Sidak’s multiple comparisons test, MR WT: *n* = 30 ears from 16 mice, MR^TMX^cKO: *n* = 21 ears from 14 mice]. Accordingly, the inter-peak latency of MR^TMX^cKO mice reached similar values in comparison to their WT controls (Supplementary Figure S2A). On the other hand, the TMX-mediated deletion of central GR resulted in slightly reduced ABR wave IV/I ratio indicating less neural gain, although this effect was not statistically significant (Figure 2B, left, RM 2-way ANOVA, *F*(1, 28) = 3.284, *p* = 0.0807, Sidak’s multiple comparisons test, GR WT: *n* = 14 ears from 8 mice, GR^TMX^cKO: *n* = 16 ears from 8 mice), despite the enhanced/disinhibited ABR wave amplitudes in these conditional mutants (Marchetta et al., 2022). The inter-peak latency of GR^TMX^cKO mice was significantly shorter in comparison to their WT controls, particularly above 30 dB (Supplementary Figure S2B). Finally, the conditional deletion of both MR and GR in the MRGR^TMX^cKOs, resulted in an unchanged ABR wave IV/I ratio (Figure 2C, left, RM 2-way ANOVA, *F*(1, 42) = 0.1546, *p* = 0.6962, MRGR WT: *n* = 19 ears from 11 mice, MRGR^TMX^cKO: *n* = 25 ears from 14 mice). We interpret this as an intermediate response resulting from counterbalancing effects of the individual cKOs. In addition, the inter-peak latency of MRGR^TMX^cKO mice was equally long as for their WT controls (Supplementary Figure S2C). As we assume that in our mouse model MR and GR are not deleted in the ascending auditory pathway (cochlea, brainstem), the alterations observed in auditory neural gain are likely a top-down effect resulting from the forebrain deletion of MR and/or GR, as also described to be the case for blood pressure changes resulting from MR overexpression under the CamKIIα promoter (Lai et al., 2007).

The predicted influence of stress and hippocampal LTP on central auditory adaptive responses (Singer et al., 2013b; Jafari et al., 2018; Matt et al., 2018; Savitska et al., 2022; Zhang et al., 2022) motivated us to study hippocampal LTP on acute coronal brain slices in the individual and compound MR and GR^TMX^cKO models (Figure 2, right). LTP was induced by tetanic stimulation (1 s, 100 Hz) of CA3 Schaffer’s collateral axons, and fEPSPs were recorded from the dendrites of CA1 pyramidal cells that form synaptic contacts with CA3 neurons (Matt et al., 2018). LTP, determined by averaging fEPSP slopes from the period between 50 and 60 min after the high-frequency stimulation (HFS), was significantly higher after 50–60 min with changes seen from ~35 min onwards in MR^TMX^cKOs (Figure 2A, right, green; 162.16% ± 8.25%, *n* = 9 slices from 5 animals) in comparison to WT controls [Figure 2A, right, gray; 125.44% ± 7.90%, *n* = 7 slices from 4 animals; Mann–Whitney *U*-test, *U*(117.8, 167.9) = 6, *p* = 0.0093], while the GR^TMX^cKOs had a trend toward lower LTP in comparison to WT controls [Figure 2B, right, GR^TMX^cKO, red, 128.96% ± 8.98%, *n* = 9 slices from 6 animals, WT, gray, 165.52% ± 18.27%, *n* = 7 slices from 5 animals; Mann–Whitney *U*-test, *U*(151.0, 124.0) = 12, *p* = 0.0721]. On the other hand, MRGR^TMX^cKOs exhibited significantly lower LTP in comparison to their respective WT controls [Figure 2C, right, MRGR^TMX^cKO, blue, 122.88% ± 8.12%, *n* = 13 slices from 7 animals, WT, gray, 157.30% ± 13.18%, *n* = 12 slices from 6 animals; Mann–Whitney *U*-test, *U*(160.7, 121.3) = 45, *p* = 0.0076].

To investigate to what extent the differences in central compensation or LTP are associated with differences in the presynaptic state of Schaffer’s collaterals in the hippocampus, we studied paired-pulse facilitation (PPF), an indication of presynaptic activity underlying short-term plasticity. PPF, a transient increase in the probability of glutamate release (Zucker and Regehr, 2002), was studied in MR^TMX^cKO, GR^TMX^cKO, and MRGR^TMX^cKO brain slices as described in methods and previous studies (Satake et al., 2012).

**Figure 3:**
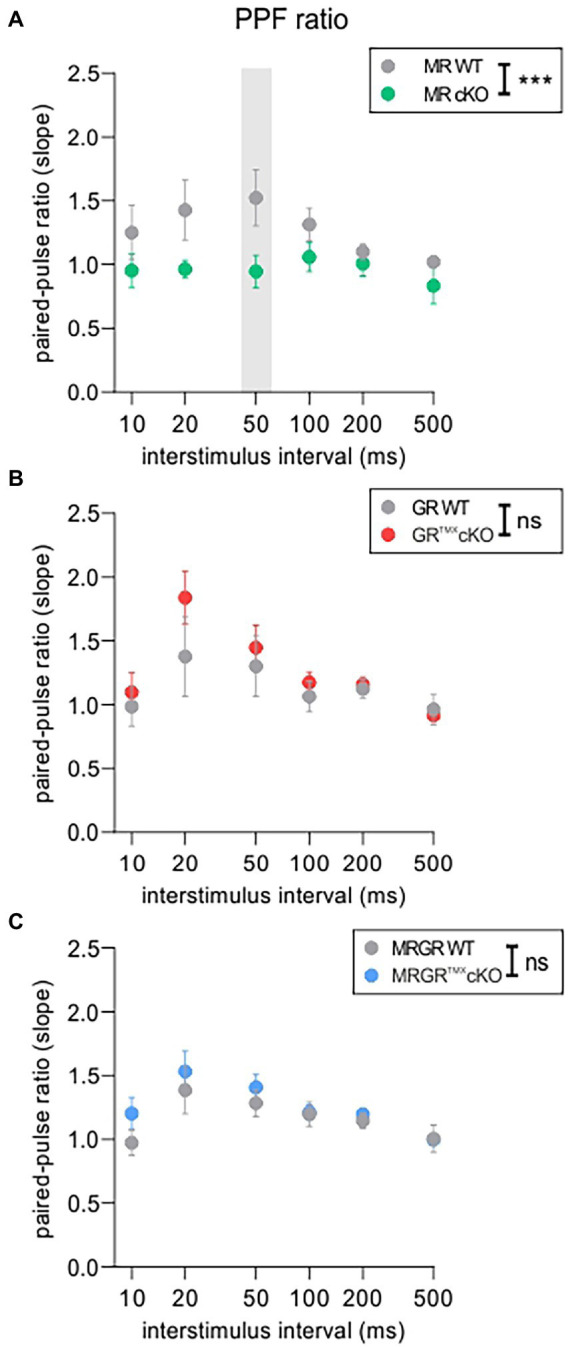
PPF as an indicator of short-term plasticity. **(A)** The analysis of PPF in MR^TMX^cKOs (green) showed a significantly lower paired-pulse ratio of the EPSP2/EPSP1 slope in comparison to WT controls (gray) with a significant post-hoc test at 50 ms ISI. **(B)** GR^TMX^cKOs (red) showed no difference in paired-pulse ratio of the EPSP2/EPSP1 slope in comparison to WT controls (gray). **(C)** MRGR^TMX^cKOs (blue) showed no difference in paired-pulse ratio of the EPSP2/EPSP1 slope in comparison to WT controls (gray). Mean ± SEM. ns = *p* > 0.08, *** = *p* < 0.001.

The PPF was investigated in each brain slice prior to LTP induction using varying ISIs of 10, 20, 50, 100, 200, and 500 ms and the same stimulation strength that was also used for LTP recordings (Figure 3). The MR^TMX^cKO mice had a significantly lower PPF, seen in its slope ratio, in comparison to their WT controls [Figure 3A, green vs. gray circles, 2-way ANOVA, *F*(1, 78) = 14.48, *p* = 0.0003, Sidak’s multiple comparisons test, MR^TMX^cKO: *n* = 8 slices from 5 animals, WT: *n* = 7 slices from 4 animals]. In contrast, in GR^TMX^cKO mice, the PPF slope ratio was not significantly different from their respective controls [Figure 3B, red vs. gray circles, 2-way ANOVA, *F*(1, 78) = 2.172, *p* = 0.1446, GR^TMX^cKO: *n* = 9 slices from 6 animals, WT: *n* = 7 slices from 5 animals]. In MRGR^TMX^cKO mice also, no difference in PPF slope ratio was observed in comparison to their WT controls [Figure 3C, blue vs. gray circles, 2-way ANOVA, *F*(1, 138) = 2.217, *p* = 0.1388, MRGR^TMX^cKO: *n* = 13 slices from 7 animals, WT: *n* = 12 slices from 6 animals].

We further examined the fEPSP slopes and fiber volley (FV) amplitudes of individual cKOs, in order to ensure that the effects observed in the LTP and PPF were not due to a change in the basal synaptic transmission properties. fEPSP slope or FV amplitudes in MR^TMX^cKOs (Supplementary Figure S3A), GR^TMX^cKOs (Supplementary Figure S3B), and MRGR^TMX^cKOs (Supplementary Figure S3C) were not different from their respective WT controls. Further, the increase in fEPSP slope remained proportional to FV amplitudes and did not differ between the different genotypes (Supplementary Figure S3A–C, bottom). Overall, the findings suggest that basal synaptic transmission properties were unchanged by cKO of MR and/or GR.

In conclusion, the reduced PPF in MR^TMX^cKO implies that MR coordinates the release probability in the presynapse of hippocampal neurons, possibly through elevation of GR expression levels. We further conclude that the enhanced LTP and ABR wave IV/I ratio in MR^TMX^cKOs are linked to the reduced suppression of GR expression levels in these mice. In that sense, GR expression levels regulate LTP and auditory neural gain. The lack of effect in GR^TMX^cKOs and MRGR^TMX^cKOs is consistent with this notion.

### Deletion of MR and/or GR in the hippocampus differentially affects NO-GC, GC-A, and Arc/Arg3.1 mRNA levels

**Figure 4:**
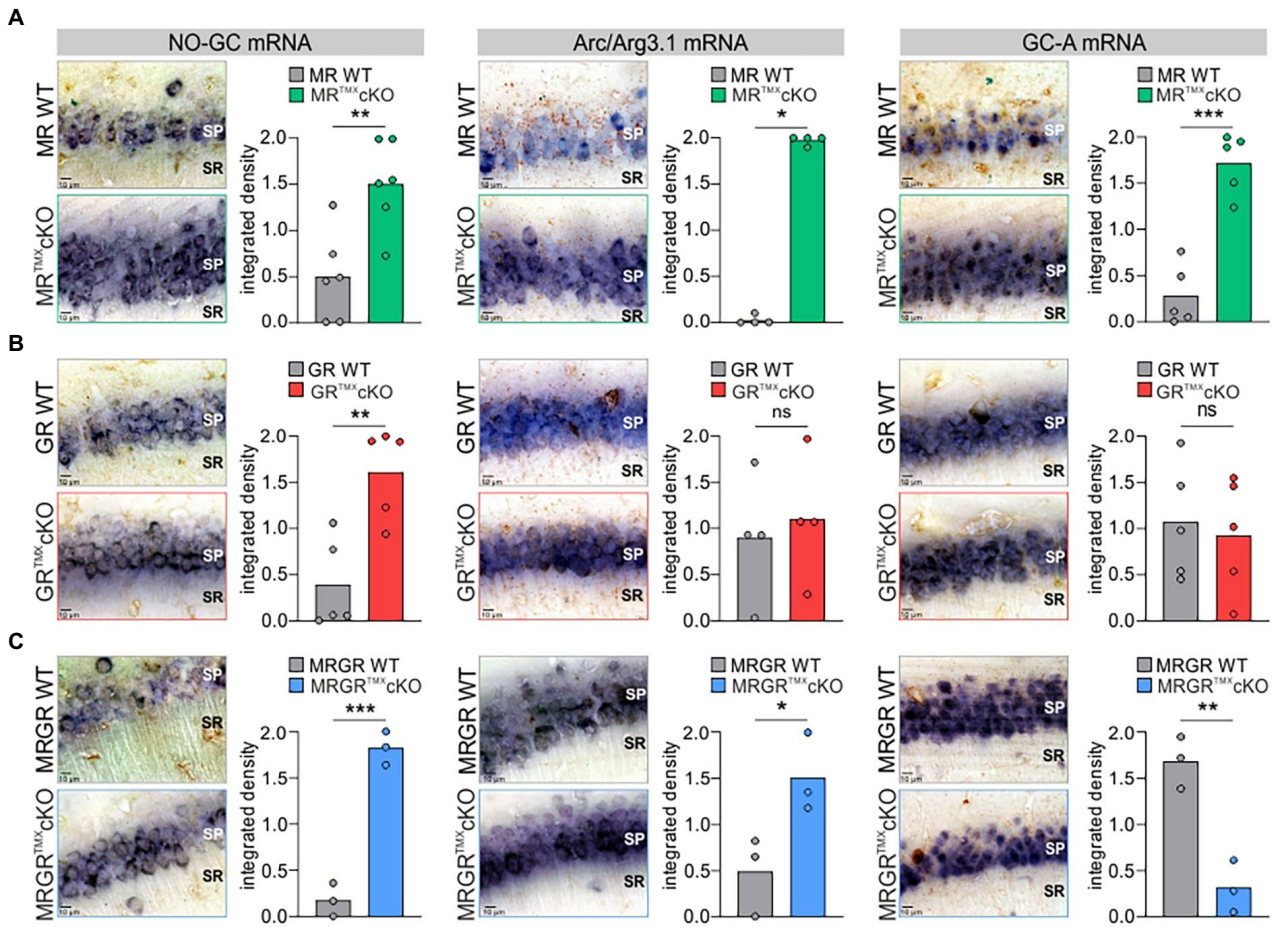
NO-GC, Arc/Arg3.1, and GC-A expression in the CA1 region of the hippocampus in MR, GR, and MRGR^TMX^cKO mice. **(A)** MR^TMX^cKO (green) mice showed significantly higher levels of NO-GC mRNA expression, Arc/Arg3.1 mRNA expression, and GC-A mRNA expression in comparison to their WT controls (gray). **(B)** GR^TMX^cKO (red) mice showed significantly higher NO-GC mRNA expression levels, equal Arc/Arg3.1 mRNA expression levels, and equal GC-A mRNA expression levels in comparison to their WT controls (gray). **(C)** MRGR^TMX^cKO (blue) mice showed significantly higher levels of NO-GC mRNA expression levels and Arc/Arg3.1 mRNA expression, but significantly lower GC-A mRNA expression levels in comparison to their WT controls (gray). Mean. ns = *p* > 0.08, * = *p* < 0.05, ** = *p* < 0.01, *** = *p* < 0.001.

To better understand the differential impact of acute deletion of MR and GR function on hippocampal LTP and auditory processing, we tested for altered cGMP generator expression profiles, previously hypothesized to provide the missing link between auditory processing and LTP. The mRNA expression profiles of a crucial NO-GC subunit and membrane-bound GC-A were analyzed in the CA1 region of the hippocampus. Two isoforms of NO-GC exist: the more widely expressed NO-GC1 and NO-GC2. Both isoforms build a heterodimer complex with the beta-1 subunit (Koesling et al., 2016), the changes of which parallel that of the alpha subunit (Mergia et al., 2006; Koesling et al., 2016). Therefore, we targeted NO-GC β1 mRNA for analysis of expression profiles. The recently observed potentiating influence of altered cGMP generator activity on LTP and synaptic AMPA receptor transport activity (Nelissen et al., 2021, 2022) moreover motivated us to correlate levels of cGMP generators with changes of cytoplasmic Arc/Arg3.1, the mRNA of which is targeted to dendrites during LTP/LTD changes in response to neuronal activity (Korb and Finkbeiner, 2011; Goel et al., 2019). *In situ* hybridizations of NO-GC β1, GC-A, and Arc/Arg3.1 mRNA were analyzed in brain sections and quantified in the CA1 region of the hippocampus according to previously established protocols (Matt et al., 2018; Eckert et al., 2021; Figure 4). MR^TMX^cKOs exhibited significantly higher levels of NO-GC, Arc/Arg3.1, and GC-A mRNA in comparison to their WT controls [Figure 4A, NO-GC left, unpaired two-tailed student’s *t*-test, *t*(10) = 3.651, *p* = 0.0045, *n* = 6 mice each; Arc/Arg3.1 mRNA middle, unpaired two-tailed student’s *t*-test, *t*(6) = 53.63, *p* < 0.0001, *n* = 4 mice each; GC-A mRNA right, unpaired two-tailed student’s *t*-test, *t*(8) = 6.874, *p* = 0.0001, *n* = 5 mice each]. GR^TMX^cKOs also had significantly higher levels of NO-GC mRNA [Figure 4B, left, unpaired two-tailed student’s *t*-test, *t*(8) = 3.924, *p* = 0.0044, *n* = 5 mice each] but strikingly showed no significant differences in levels of Arc/Arg3.1 and GC-A mRNA in comparison to their WT controls [Figure 4B, Arc/Arg3.1 mRNA middle, unpaired two-tailed student’s *t*-test, *t*(6) = 0.4093, *p* = 0.6965, *n* = 4 mice each; GC-A mRNA right, unpaired two-tailed student’s *t*-test, *t*(8) = 0.3671, *p* = 0.7231, *n* = 5 mice each]. MRGR^TMX^cKO displayed significantly higher levels of NO-GC mRNA and Arc/Arg3.1 mRNA, as also observed in MR^TMX^cKOs [Figure 4C, NO-GC, left, unpaired two-tailed student’s *t*-test, *t*(4) = 11.17, *p* = 0.0004, *n* = 3 mice each; Arc/Arg3.1 mRNA middle, unpaired two-tailed student’s *t*-test, *t*(4) = 2.883, *p* = 0.0449, *n* = 3 mice each]. On the other hand, the GC-A expression levels in the MRGR^TMX^cKO were significantly reduced [Figure 4C, right, unpaired two-tailed student’s *t*-test, *t*(4) = 5.941, *p* = 0.0040, *n* = 3 mice each].

We additionally tested for altered cGMP generator expression levels in the auditory cortex of MR^TMX^cKO and GR^TMX^cKO mice in neurons of all cortical layers (layer I–VI). We found that GC-A expression levels in the auditory cortex for both MR^TMX^cKO and GR^TMX^cKO mice mirror the findings in the hippocampus (Supplementary Figure S4), hypothalamus, and amygdala (data not shown). NO-GC expression levels in MR^TMX^cKO and GR^TMX^cKO mice were also both significantly higher in the auditory cortex in comparison to their respective WT controls (Supplementary Figure S4), as also observed in the hippocampus; however, NO-GC levels throughout the rest of the brain were more variable (data not shown).

In order to test whether MR or GR directly influence NO-GC or GC-A *via* their classical transcriptional cis-activation GR element binding motifs, a search was carried out for MR/GR-specific binding motifs (GRE sequences) in the genes encoding NO-GC (i.e., *Gucy1a1, Gucy1a2, Gucy1b1*) and GC-A (*Npr1*). No relevant GRE sequences could be detected in *Gucy1b1* or *Npr1*. For *Gucy1a1* and *Gucy1a2*, each gene was found to contain a conserved GRE sequence (Supplementary Figures S5, S6). Specifically, for *Gucy1a1*, this GRE sequence (5′-AGGAACACCATGTTCTG-3′) was found to span across the start codon of the transcribed part of the gene. This also included an Atoh sequence (5′-CAGAAGG-3′) 25 bp upstream of the GRE sequence. For *Gucy1a2*, the GRE sequence was a palindrome (5′-AGAACAAACTGTTCT-3′) located near multiple regulatory regions in the intron between exons 3 and 4.

**Figure 5:**
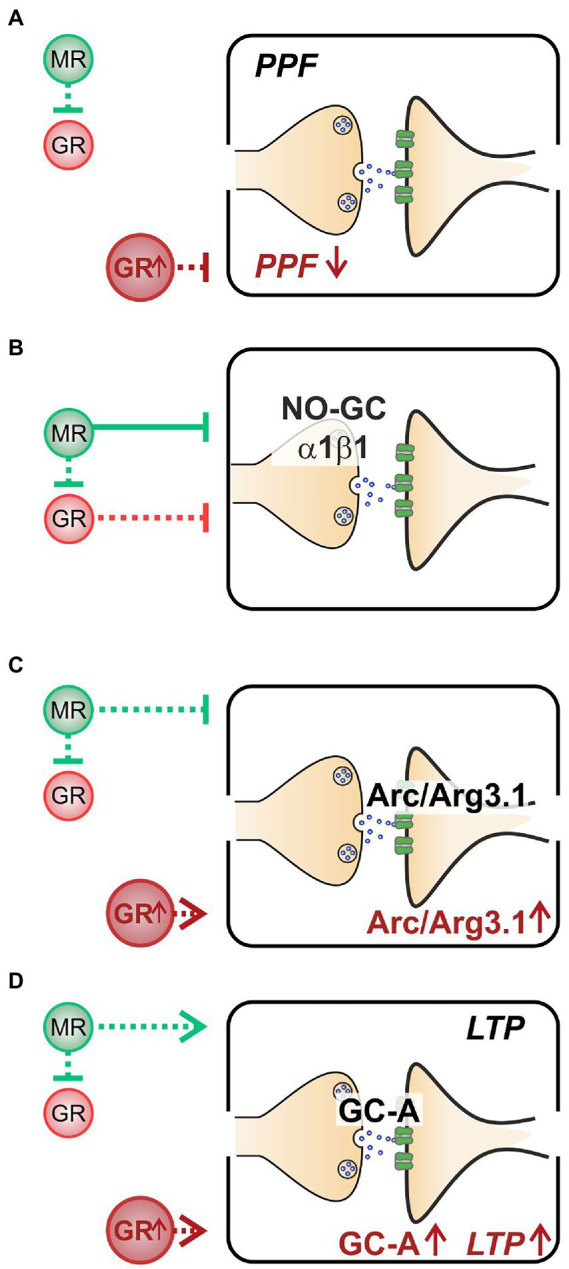
A summary of the hypothesized effects of MR and GR in a physiological state and stress state (GR↑). **(A)** When at a normal level, MR inhibits GR (green dashed line, upper half). However, neither MR nor GR affect PPF when at physiological levels. At elevated GR conditions (lower half), PPF is low. **(B)** Both stress receptors exhibit an inhibitory effect on NO-GC expression levels, though the inhibitory effect of MR (green solid line) is stronger than that of GR (red dashed line). **(C)** MR inhibits Arc/Arg3.1 expression levels (green dashed line, upper half); however, when GR is elevated, Arc/Arg3.1 is stimulated (lower half). **(D)** Basal MR stimulates GC-A expression levels and LTP (green dashed line, upper half). Elevated GR expression also leads to elevated GC-A and to elevated LTP (lower half).

**Figure 6:**
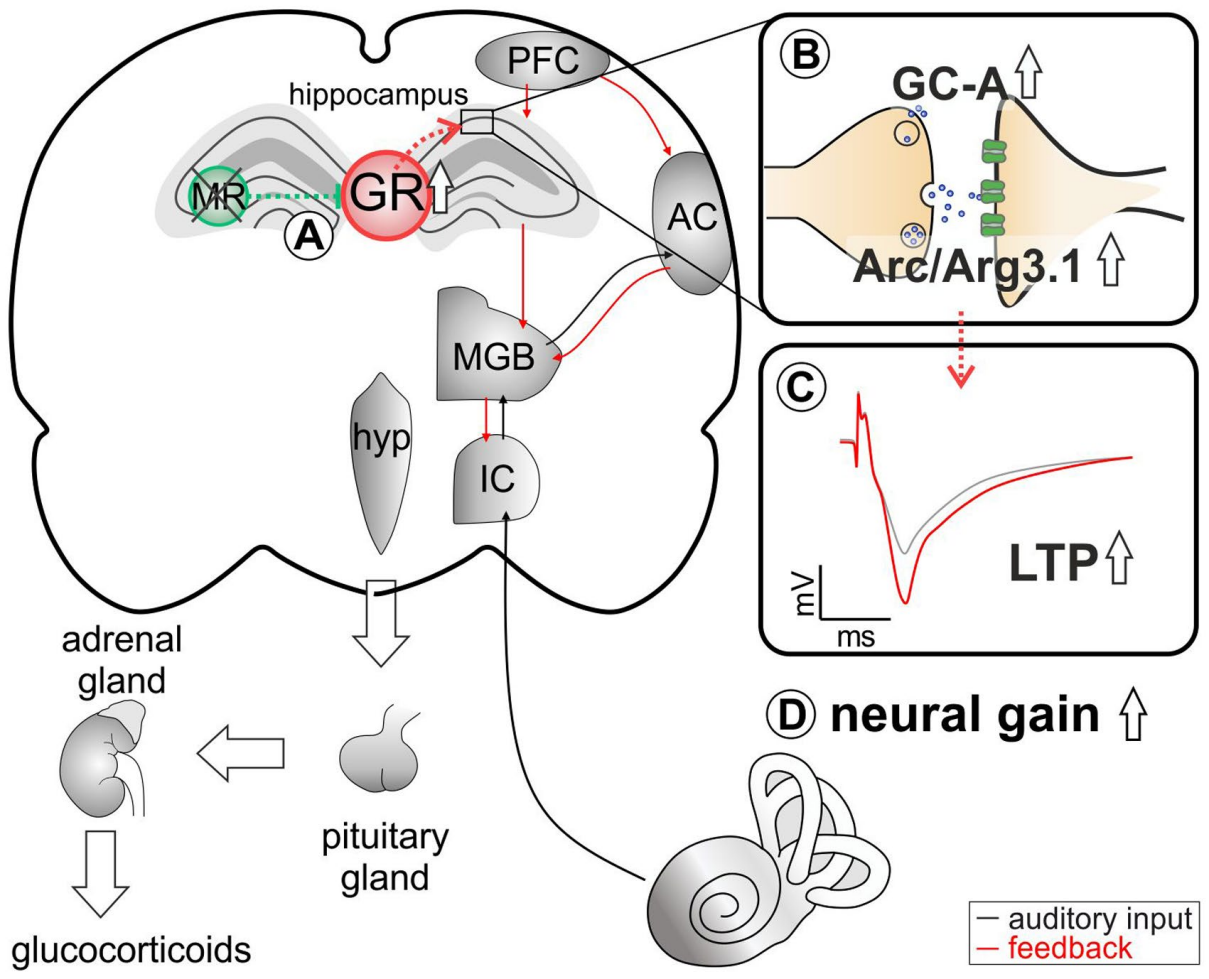
An abstract scheme of the pathways that can be concluded from the acute deletion of MR (green). **(A)** MR deletion leads to elevated GR expression levels. **(B)** As a result of elevated GR, an increase in GC-A and Arc/Arg3.1 expression levels in the hippocampus can be observed. **(C)** This change in expression pattern may be functionally related to higher hippocampal LTP. **(D)** Elevated GC-A and Arc/Arg3.1 expression levels and elevated LTP potentially provide a means for top-down mechanisms allowing for auditory neural gain.

Altogether, we here demonstrate that (i) decreased PPF in MR^TMX^cKOs may be linked to elevated GR expression levels (Figure 5A). (ii) Enhanced NO-GC expression levels in MR, GR, and MRGR^TMX^cKOs, imply that both receptors’ activity is required to effectively suppress NO-GC in hippocampal neurons under physiological conditions, which may be supported by GRE elements responding to both MR and GR (Figure 5B). (iii) Increased Arc/Arg3.1 expression levels in MR^TMX^cKOs and MRGR^TMX^cKOs but not in GR^TMX^cKOs (Figure 5C) do not correlate to LTP as simply. The changes of Arc/Arg3.1 in MRGR^TMX^cKOs rather point to basal MR activity but not GR activity as being inhibitory to Arc/Arg3.1 levels (Figure 5C). Further, the gradient of Arc/Arg3.1 changes in MR^TMX^cKOs and MRGR^TMX^cKOs suggests that elevated GR expression may stimulate Arc/Arg3.1 (Figure 6B). (iv) MR^TMX^cKOs exhibited an increased GR expression, suggesting that under physiological conditions MR suppresses GR expression. When this suppression is removed in MR^TMX^cKOs, animals exhibit elevated LTP, GC-A, and ABR wave IV/I ratio. This is not seen in GR^TMX^cKOs, suggesting that GR may contribute to the empowerment of central neural gain during auditory adaption by controlling the energization through GC-A (Figure 5D).

## Discussion

By investigating TMX-induced conditional single or combined deletion of MR and GR in forebrain regions, we show for the first time that central MR and GR activity must be balanced in order to set thresholds for presynaptic activity and central auditory compensation (auditory neural gain). Changes in MR and GR activities result in significant alterations of cGMP generator expression levels in hippocampal neurons. In detail, we suggest (i) baseline hippocampal MR expression levels suppress GR expression. (ii) MR-induced control of GR may set a threshold for presynaptic plasticity. (iii) Basal MR may set a threshold for AMPA receptor trafficking by Arc/Arg3.1, while elevated GR levels may enhance Arc/Arg3.1 expression levels. (iv) Elevated GR may enhance LTP and GC-A expression levels and thereby contribute to LTP-dependent neural auditory gain.

### Basal MR activities suppress GR expression

Until now, insights into the physiological function of stress receptors in the adult brain have been limited because global GR KOs (Cole et al., 1995) or early Cre-mediated deletion of GR (Erdmann et al., 2008) is lethal in mice. Also, a global lack of MR induces early postnatal death linked with bodily dehydration (exsiccosis) due to massive renal sodium and water loss (Berger et al., 1998). Early Cre-induced conditional MR deletion is expected to allow long-term adaptation processes to compensate for the absence of MR (Erdmann et al., 2008). Some of these limitations were avoided by the acute TMX-induced deletion of MR and GR studied here. Although in Marchetta et al., 2022, a TMX-induced deletion of MR and GR protein was only shown for the MRGR^TMX^cKO and not for the single MR^TMX^cKO and GR^TMX^cKO, the observed higher GR expression levels in MR^TMX^cKOs, also previously reported in global or conditional MR^TMX^cKO mice (Berger et al., 2006; Erdmann et al., 2007), may point to successful targeting of individual receptor expression. We however cannot entirely exclude that the receptor protein may not have been completely deleted (knocked out), at the time of the experiments. Nevertheless, it can be assumed that an upregulation of GR in MR^TMX^cKOs reflects the previously-described balancing effect of MR on the hypothalamic-pituitary-adrenal (HPA) axis, which stems from central neurons (see for a review de Kloet et al., 2018). Thus, elevated corticosterone levels were reported in conditional GR but not MR mutants (Erdmann et al., 2007), and consistently higher corticosterone levels were measured in GR^TMX^cKOs but not in MR^TMX^cKOs (Marchetta et al., 2022). In line with this, normal corticosterone levels—occurring when HPA axis feedback regulation is balanced—are the result of physiologically lower GR levels when baseline MR activities prevent excessive GR expression (Harris et al., 2013), as also concluded from the findings of the present study. This tight control of the HPA axis was recently demonstrated in heterozygous GR mutants that had an enhanced HPA axis activity in response to restraint stress (Harris et al., 2013). Interestingly, the HPA axis overshoot in heterozygous GR mutants in response to stress was counterbalanced through an overexpression of MR in these heterozygous GR mutants (Harris et al., 2013). Apparently, MR exerts a tonic inhibitory influence on HPA axis activity, and thereby determines the threshold of reactivity during stress (see for a review de Kloet et al., 2018).

In conclusion, we suggest that the predicted tonic inhibitory influence of MR on the HPA axis is severely disturbed upon the acute MR or GR deletion.

### GR may suppress plasticity in presynaptic hippocampal activity

The TMX-induced deletion of MR in hippocampal regions of MR^TMX^cKOs resulted in reduced PPF (Figures 3, 5A). Because PPF results from a prior accumulation of residual Ca^2+^ at the synaptic terminal and a lingering effect of Ca^2+^ on the exocytotic Ca^2+^ sensor of releasable vesicles during the second stimulus (as reviewed in Thomson, 2000; Zucker and Regehr, 2002), we expect the probability of vesicular release to be transiently decreased in MR^TMX^cKOs. This is likely due to increased GR expression in these mutants, as can be concluded from the absence of PPF effects in GR^TMX^cKO and MRGR^TMX^cKO (Figure 5A). We thus assume that baseline MR or GR activity alone do not strongly affect presynaptic excitability, but rather that increased GR activity, as occurs in the MR^TMX^cKOs, suppresses PPF (Figures 1A, 3A, 5A). This finding may contrast findings in which corticosterone application in hippocampal CA1 neurons resulted in a brief increase in miniature excitatory postsynaptic current frequency, a feature described to occur through MR activities (Sarabdjitsingh et al., 2016; de Kloet et al., 2018; Joels, 2018). Further studies are required to clarify this controversy. It is noteworthy to consider the findings in our previous study, which implied a reduced activity at the inner hair cell synapse in the MR^TMX^cKO (Marchetta et al., 2022). The peripheral depression of activity in MR^TMX^cKOs is comparable to the here-observed more central changes of reduced release probability in the presynapse of hippocampal neurons (Figure 3; Marchetta et al., 2022). Overall, it gives credence to the hypothesis that balanced central MR and GR activities set the threshold for sensory activity centrally and peripherally by targeting presynaptic mechanisms, possibly through tuning reactivity of top-down auditory feedback loops, which would need to be examined in future studies.

### Baseline MR and GR activity keeps NO-GC expression levels low in neurons

It was previously suggested that the complementary functions of MR and GR on presynaptic excitability in hippocampal brain neurons may be the result of distinct transcriptional networks activated by the glucocorticoid receptors (Reul and de Kloet, 1985; de Kloet et al., 2000; Obradovic et al., 2004; Mifsud and Reul, 2016; McCann et al., 2021).

Considering a possible MR/GR driven transcriptional control of pre- or post-synaptic activity by NO-GC, we searched for respective GREs that bind to MR and GR (Shimba and Ikuta, 2020) in the upstream regions of NO-GC subtypes (Shimba and Ikuta, 2020). GREs constitute a palindromic consensus sequence AGAACAnnnTGTTCT (Polman et al., 2013); however, several other GRE-like sequence motifs have been identified (Meijsing et al., 2009; van Weert et al., 2017). We found a GRE in the start codon region of *Gucy1a1* but not in the β1 subunit. In addition to a GRE sequence in *Gucy1a1,* the detection of a 25 bp Atoh sequence moreover suggests that this GRE binding motif is potentially used as a MR-specific recognition site, since GRE sequences that coincide with an Atoh consensus sequence within 400 bp of the GRE have been defined to be MR-specific (van Weert et al., 2017). Additionally, a GRE motif was found in *Gucy1a2* in an intron between exons 3 and 4, apparently completely outside of known regulatory regions, questioning the significance of this potential GRE motif. Regarding the observation of elevated *Gucy1b1* NO-GC expression levels both in single MR^TMX^cKOs and GR^TMX^cKOs as well as in double MRGR^TMX^cKOs, we must question how a potential GRE in the start codon region of *Gucy1a1*, but not β1 subunit may be interpreted in the context of the findings in the present study. While it is necessary to confirm expression changes of *Gucy1a1* in MRGR^TMX^cKOs in future studies, it is important to consider that changes in β1 subunits went along with changes in α1 expression (Mergia et al., 2006; Koesling et al., 2016). Further, expression levels of NO-GC1 and NO-GC2 in the brain mirror each other (Koesling et al., 2016). Thus, we hypothesize that under physiological conditions, NO-GC1 (α1, β1) or NO-GC2 (α2, β1) is suppressed by MR and GR (Figure 5B). The elevation of NO-GC expression in MRGR^TMX^cKOs in comparison to MR^TMX^cKOs, which exhibit elevated GR expression levels. This may suggest that the inhibitory effect of MR on NO-GC may outweigh that of GR. Using NO-GC1 and NO-GC2 KO mice, the differential localization of NO-GC1 in hippocampal presynapses and NO-GC2 in postsynapses was shown to contribute to LTP through facilitation of presynaptic (NO-GC1) and postsynaptic (NO-GC2) excitability of hippocampal neurons (see for a review Koesling et al., 2016). Although more detailed studies are needed to validate the functionality of the GRE binding motifs, the data nevertheless suggest one possible pathway of how MR and GR could influence NO-GC1 (Figure 5B). This is in line with the observation that steroid hormone receptors exert positive or negative effects on the expression of target genes (Beato and Klug, 2000). Thereby, our findings suggest that baseline MR and GR activity could control the NO-GC abundance, which may be relevant for sensing NO released from eNOS- and nNOS-producing hippocampal cells to influence plasticity events (Son et al., 1996; Hopper and Garthwaite, 2006).

In conclusion, baseline MR and GR activity may keep NO-GC expression levels in neurons low. The target of a specific GRE element found in upstream regions of *Gucy1a1* NO-GC1, the dominant NO-GC isoform in hippocampal presynapses, may need further specification.

### Baseline MR may keep Arc/Arg3.1 levels low in neurons

TMX-induced deletion of MR in frontal brain regions resulted in increased Arc/Arg3.1 expression levels in the hippocampus (Figure 4A). As Arc/Arg3.1 expression levels in the hippocampus are elevated in MRGR^TMX^cKOs but not GR^TMX^cKOs (Figure 4C), we hypothesize that basal activity of MR but not GR keeps Arc/Arg3.1 expression levels in hippocampal neurons low (Figure 5C). While we hypothesize that basal levels of GR have no effect on Arc/Arg3.1 expression levels, higher levels of GR (as seen in MR^TMX^cKOs) enhance it (Figure 6B).

Arc/Arg3.1 expression changes have been shown to influence the strength of individual synapses, during both LTP (Rodriguez et al., 2005) and LTD (Guzowski et al., 1999; Plath et al., 2006; Tzingounis and Nicoll, 2006; Park et al., 2008; Waung et al., 2008; Bramham et al., 2010; Yilmaz-Rastoder et al., 2011; Wall and Correa, 2018; Zhang and Bramham, 2021). Moreover it is important to consider that neuronal stimuli induce the rapid transcription of the Arc/Arg3.1 gene (within 5 min; Ramirez-Amaya et al., 2005) and translocation of its mRNA from the nucleus to the cytoplasm (within 30 min; Guzowski et al., 1999). From the cytoplasm, Arc/Arg3.1 mRNA incorporates into a large ribonucleoprotein complex that is actively transported along the dendrite (Dynes and Steward, 2007). From the pre-existing Arc/Arg3.1 mRNA pool in dendrites, a selected pool is translated rapidly by acute neuronal activity, changing surface expression of AMPA receptors (Hedde et al., 2021). The present study may indicate that baseline MR levels influence the level of Arc/Arg3.1 mRNA in the cytoplasm of pyramidal neurons. In addition, when the control of GR expression by MR is lost (as in MR^TMX^cKOs), Arc/Arg3.1 expression levels are enhanced more than in MRGR^TMX^cKOs. This indicates that elevated GR levels may stimulate the level of Arc/Arg3.1 mRNA in the cytoplasm of pyramidal neurons. This makes sense considering that previous studies demonstrated that a selective activation of glucocorticoid receptors promotes lateral diffusion and enhanced surface expression of AMPA receptors in CA1 neurons (Karst and Joels, 2005; Groc et al., 2008; Martin et al., 2009), a feature linked with LTP (Groc et al., 2008). It is therefore feasible that MR—both at basal levels and through control of GR—sets the reactivity for Arc/Arg3.1 mobilization in dendrites and its rapid translation in spines through defining the cytoplasmatic Arc/Arg3.1 mRNA pool.

### Elevated GR activity relates to elevated LTP, elevated GC-A, and elevated auditory neural gain

A strong association has been suggested between critical auditory nerve activity and central auditory neural gain, measured *via* enhanced ABR wave IV/I ratio, and plasticity changes in LTP, assessed both through *in vitro* electrophysiology (Marchetta et al., 2020b; Savitska et al., 2022) and through a hippocampus-dependent learning test in animal models (Matt et al., 2018). It has been speculated that a critical input of auditory activity is necessary for the recruitment of a reinforcement process that interacts between the auditory thalamus, prefrontal cortex (PFC), and the hippocampus. Thus, the dorsal aspect of the MGB, which receives input from the primary auditory cortex and projects to higher-order auditory regions (Antunes and Malmierca, 2021; Mease and Gonzalez, 2021), is likely part of the topographically complex connectivity pattern projecting between the medio-dorsal thalamus, the medial PFC, and the hippocampal formation (Bueno-Junior and Leite, 2018). These corticofugal projections from the PFC influence auditory processing at lower levels of the cortical sensory hierarchy and often include activation of mesolimbic areas, such as the basolateral amygdala, the activation of which is involved in top-down feedback reinforcement processes (Asilador and Llano, 2020; Suga, 2020).

The medio-dorsal thalamus/PFC/hippocampal connectivity (see Figure 6) is not only sensitive to stress responses (for review, see Jett et al., 2017), but is also part of the extra-hypothalamic pathways involved in stress-control, emotional states, attention, and vigilance influenced by glucocorticoids (de Kloet et al., 2000, 2019; Wingenfeld and Otte, 2019; Antunes and Malmierca, 2021; Knipper et al., 2022). Interestingly, the TMX-induced deletion of MR in frontal brain regions resulted in enhanced ABR wave IV/I ratio (neural gain) and LTP. If this response were due to increased GR expression resulting from the loss of MR, an acute deletion of GR should result in opposite changes of ABR wave IV/I ratio and LTP. In line, when GR is deleted (as in GR^TMX^cKOs and MRGR^TMX^cKOs), animals do not have higher wave IV/I ratio and their LTP is unchanged or lower, respectively, indicating that GR elevation – but not baseline GR levels – contributes to elevated neural gain and LTP. Interestingly, the higher wave IV/I ratio and LTP in MR^TMX^cKO occurred with an elevation of GC-A expression levels. Also, lower LTP occurred with lower GC-A, as can be seen from MRGR^TMX^cKOs (Figure 5D). Comparable changes in GC-A were detected not only in the hippocampus but also in the auditory cortex (Supplementary Figure S4) and amygdala. Differences in GC-A activity may thus provide a means for altering corticofugal top-down feedback facilitation processes (Asilador and Llano, 2020; Suga, 2020) and thereby contribute to auditory neural gain.

Oitzl and de Kloet were the first to demonstrate that MRs and GRs mediate the storage of spatial information in a coordinated manner (Oitzl and de Kloet, 1992). Their study suggested that MR promotes the extinction of information, while GR is essential for consolidation. Supporting this, memory storage was impaired when progesterone and the glucocorticoid antagonist mifepristone was given immediately after the learning trial (Oitzl and de Kloet, 1992), while the MR antagonist spironolactone under the same condition did not affect consolidation but rather retrieval (Oitzl and de Kloet, 1992). Meanwhile, memory storage was shown to be impaired in numerous studies, e.g., when GRs are deleted in the amygdala or hippocampus or when GR antagonists are administered in the hippocampus immediately after learning, prior to consolidation (see for a review de Kloet et al., 1999; Rodrigues et al., 2009; Luksys and Sandi, 2011; Roozendaal and McGaugh, 2011; Schwabe et al., 2012; de Kloet et al., 2018).

Very recently, the treatment of WT mice with a phosphodiesterase 9A inhibitor restored a stress-induced drop of temporal auditory processing and hippocampal LTP (Savitska et al., 2022). This finding can now possibly be linked to elevated auditory neural gain, LTP, and GC-A expression levels. Increased GC-A expression levels here corresponded with elevated GR expression levels when the inhibition of GR was removed in MR^TMX^cKOs, while expression levels were not elevated in GR^TMX^cKOs (Figure 4). Indeed, the memory-enhancing phosphodiesterase 9A inhibitor (Kroker et al., 2012) has been suggested to regulate a pool of cGMP that is independent of nNOS (Harms et al., 2019). Other findings suggest that the phosphodiesterase 9A in the brain even preferentially regulates nuclear- and membrane-proximal pools of cGMP, which include the GC-A generated cGMP pools (Patel et al., 2018). Within this framework, the present study links for the first time the recognized function of the GR stress receptors in LTP (de Kloet et al., 2018; Joels, 2018) with the perfusion and blood supply driven metabolism-promoting function of the transmembrane GC-A. GC-A is activated through the atrial natriuretic peptide and brain natriuretic peptide (Potter, 2011) and has meanwhile emerged as a key regulator of energy consumption and metabolism counteracting vasoconstriction by inducing vasodilation (Kuhn, 2016). This role of GC-A has also been linked with neurogenesis (Muller et al., 2009) and angiogenesis (Kuhn et al., 2009), two processes which are essential for proper memory-dependent processes (Anacker and Hen, 2017). A critical role of GC-A for central auditory processing has been suggested through the use of global GC-A KO mice, which are unable to maintain proper central auditory processing following auditory trauma (Marchetta et al., 2020a).

## Conclusion

Our data suggest that under physiological conditions, MR suppresses GR expression to keep the threshold for memory consolidation high (Figure 6A). The threshold of LTP is implemented *via* MR-induced control of postsynaptic Arc/Arg3.1 levels for optimized AMPA-receptor trafficking (Figure 6B), *via* MR- and GR-induced control of NO-GC (Figure 5B) and neuronal GC-A levels (Figure 6B) for optimized energy supply during hemodynamic responses.

Hemodynamic responses take place within a glutamatergic neural feedforward signaling, that includes a neuronal-derived nitric oxide (NO) release from the glutamatergic synapses as well as endothelial-derived NO from blood vessels (Sohal et al., 2009; Czeh et al., 2015; Lee et al., 2015; Chen et al., 2017; Csabai et al., 2018; Czeh et al., 2018; Han et al., 2019). This is followed by a metabolic feedback signal in the smooth muscle cells of parenchymal arterioles, which results in vasodilation (for a review see Attwell et al., 2010; Kisler et al., 2017; Ledo et al., 2021). cGMP signaling plays an integral role for the LTP results of the present study (Sanderson and Sher, 2013; Bradley and Steinert, 2016; Dorner-Ciossek et al., 2017; Borovac et al., 2018). We conclude that GR inhibition by MR may define the threshold for hemodynamic responses through the control of GC-A levels (Figure 6B). Through lowering GR expression levels *via* MR, the threshold for metabolically-demanding neuronal responses and vasodilation (possibly requiring GC-A) would be kept high. In future studies, it would be of particular interest to investigate how the suppression of NO-GC by MR and GR and the elevation of GC-A by increased GR levels contribute to LTP and related auditory neural gain (Figure 6).

### Limitations of the study

The search for GRE- and MR-specific binding motifs requires experimental validation of the functionality of the binding sites. Moreover, the observation that the *in silico* prediction tools did not identify GRE motifs in the promoter regions of GC-A or Arc/Arg3.1 does not rule out their existence. Adequate validation of the function of GC-A for LTP-dependent auditory processing and its explicit relation to balanced stress levels is also pending and requires inducible deletion of GC-A under conditions comparable to those shown here for MR/GR deletion.

## Data availability statement

The datasets generated and/or analyzed during the current study are available from the corresponding author upon reasonable request.

## Ethics statement

The animal study was reviewed and approved by University of Tübingen, Veterinary Care Unit and the Animal Care and Ethics Committee of the regional board of the Federal State Government of Baden-Württemberg, Germany. Written informed consent was obtained from the owners for the participation of their animals in this study.

## Author contributions

MK and LR conceived the study. DC, MH, PM, JM, and WS performed the experiments and analyzed the data. EN and JP performed data sequencing analysis. MK, LR, RL, PR, JP, and PS wrote the manuscript. MK, WS, and LR supervised the work. MK, LR, RL, PR, PS, EN, and JP reviewed and edited the manuscript. All authors contributed to the article and approved the submitted version.

## Funding

This work was funded by the Deutsche Forschungsgemeinschaft (DFG, German Research Foundation) – DC, PM, MK, RL, and PR are members of the Research Training Group [grant number 335549539/GRK 2381]; FOR 2060 project RU 713/3-2, SPP 1608 RU 316/12-1, KN 316/12-1. JM was supported by the Interdisziplinäres Promotionskolleg Medizin of the University of Tübingen. EN and JP are supported by a restricted research grant from BAYER. Each source of funding provided a budget for personnel and material costs. We acknowledge support by Open Access Publishing Fund of University of Tübingen. BAYER was not involved in the study design, collection, analysis, interpretation of data, the writing of this article, or the decision to submit it for publication.

## Conflict of interest

PS was employed by the company BAYER.

The remaining authors declare that the research was conducted in the absence of any commercial or financial relationships that could be construed as a potential conflict of interest.

## Publisher’s note

All claims expressed in this article are solely those of the authors and do not necessarily represent those of their affiliated organizations, or those of the publisher, the editors and the reviewers. Any product that may be evaluated in this article, or claim that may be made by its manufacturer, is not guaranteed or endorsed by the publisher.

## Abbreviations

Arc/Arg3.1: activity regulated cytoskeletal protein
ABR: auditory brainstem response
BW: body weight
cGMP: 3′,5′-cyclic guanosine monophosphate
FV: fiber volley
fEPSP: field excitatory postsynaptic potential
GC: glucocorticoids
GR: glucocorticoid receptor
GRE: glucocorticoid response element
GR^TMX^cKO: GR^CaMKIIαCreERT2^ knockout
GC-A: guanylyl cyclase A
HFS: high-frequency stimulation
HPA: hypothalamus-pituitary–adrenal
ISI: interstimulus interval
LTD: long-term depression
LTP: long-term potentiation
MR: mineralocorticoid receptors
NO-GC: nitric oxide (NO)-sensitive (soluble) guanylyl cyclase
MR^TMX^cKO: MR^CaMKIIαCreERT2^ knockout
MRGR^TMX^cKO: MRGR^CaMKIIαCreERT2^ knockout
PPF: paired-pulse facilitation
SEM: standard error of the mean
TMX: tamoxifen
