# Supplementary material for: Acute deletion of the central MR/GR steroid receptor correlates with changes in LTP, auditory neural gain, and GC-A cGMP signaling
Source: Front Mol Neurosci. 2023 Feb 17;16:1017761. doi: 10.3389/fnmol.2023.1017761 (PMC9983609; doi:10.3389/fnmol.2023.1017761)
Supplement: Supplementary file 1 [file Data_Sheet_1.docx]

**Supplementary Methods**

The search sequences used to identify potential GRE binding motifs can be found in Supplementary Table 1, including the search result. All gene sequences were taken from the Ensembl database, GRCm39 at the following chromosomal regions: *Gucy1a1* 3:81,998,667-82,054,163; *Gucy1a2* 9:3,525,539-3,901,975; *Gucy1b1* 3:81,938,460-81,982,849; *Npr1* 3:90,357,593-90,377,799; *Arc* 15:74,540,863-74,551,199.

| **Sequence** | **Based on** | **Result** |
| --- | --- | --- |
| 5’-gaGAGAAGATTCTGTTCTAAt-3’  5’-agAAGAACAGAATGTCCTAGg-3’  5’-ggGGAAACATTATGTACTTAt-3’  5’-aaGGAGACATTCTGTCCCATa-3’  5'-atCAGAGCAGTTTGTTCTGTt-3'  5’-ccCGGATCAAAACGTTCCGTc-3’  5’-cAGGAAGGTCACGTCCAAGg-3’  5’-actAGAACAAACAAGTCCTGC-3’  5’-atGAGTACATACCTAAGGGAc-3’ | JASPAR binding site information | Not found in *Gucy1a1, Gucy1a2, Gucy1b1, Npr1* |
| 5’- AGAACANNNTGTTCT-3’ | Polman JA et al. Datson NA 2013, Endocrionology | Found:  *Gucy1a2* between exons 3 and 4  GRCm39 9:3,606,249-3,606,265 |
| 5’-nAGnACAnnnTGTnCTn-3’ | Sequence logos from JASPAR, PMC2777810, Van Weert 2017 | Found:  *Gucy1a2* between exons 3 and 4  GRCm39 9:3,606,249-3,606,265 |
| 5’- nGGnACAnnnTGTnCCn-3’ | Sequence logos from JASPAR, PMC2777810, Van Weert 2017 | Found:  *Gucy1a1* exon 2 at the start codon. 25 bp downstream of Atoh sequence  GRCm39 3: 82,026,445- 82,026,461 reverse |
| 5’-nAGnACAnnnTGTnCCn-3’ | Sequence logos from JASPAR, PMC2777810, Van Weert 2017 | Found:  Complement strand of *Gucy1a1* exon 2 at the start codon. Indicates near-palindromic sequence  GRCm39 3: 82,026,445- 82,026,461 forward |
| 5’-nGGnACAnnnTGTnCTn-3’  5’-nGAAACAnnnTGTTCnn-3’  5’-nGGAACAnnnTGTTCnn-3’ | Sequence logos from JASPAR, PMC2777810, Van Weert 2017 | Not found |

**Supplementary Figure Legends**

**Figure S1.** Detailed experimental timeline and methods. (A) After reaching adulthood of eight weeks old, animals were evaluated for hearing function. After hearing function was confirmed, they were injected with tamoxifen (TMX) for five days. TMX injection led to a deletion of MR and/or GR in the frontal brain regions. Animals rested in home cages for four weeks, after which, hearing measurements were taken and finally tissue was collected. Adapted from (Marchetta et al., 2022). (B) Scheme of hippocampus position used for histology. Red box indicates location of pictures taken for quantification at 60x magnification. (C) Scheme of hippocampus and electrode positions used for LTP. DG = dentate gyrus; MF = mossy fiber; SL = stratum lucidum; SP = stratum pyramidale; SR = stratum radiatum**.**

**Figure S2.** Inter-peak latency. (A) MR^TMX^cKO mice reached similar values in inter-peak latency in comparison to MR WT mice. (B) GR^TMX^cKO mice had significantly shorter inter-peak latency in comparison to GR WT mice. (C) MRGR^TMX^cKO mice had equally long inter-peak latency in comparison to MRGR WT mice. Mean ± SEM. ns = p > 0.08, ** = p < 0.01, *** = p < 0.001.

**Figure S3.** Input-output relationship between fEPSP slope, fiber volley amplitude, and stimulus intensity. (A) Top, representative traces of fEPSPs with increasing stimulus intensities (from 25 µA to 150 µA in 25 µA steps). In MR^TMX^cKOs, no significant differences were found in fEPSP slope or fiber volley amplitude, and the changes in fEPSP slopes correlated with changes in fiber volley amplitudes (simple linear regression analysis, comparison between slopes of lines), compared to their WT controls. (B) Top, representative traces of fEPSPs with increasing stimulus intensities (from 25 µA to 150 µA in 25 µA steps). In GR^TMX^cKOs, no significant differences were found in fEPSP slope or fiber volley amplitude, and the changes in fEPSP slopes correlated with changes in fiber volley amplitudes (simple linear regression analysis, comparison between slopes of lines) compared to their WT controls. (C) Top, representative traces of fEPSPs with increasing stimulus intensities (from 25 µA to 150 µA in 25 µA steps). In MRGR^TMX^cKOs, no significant differences were found in fEPSP slope or fiber volley amplitude, and the changes in fEPSP slopes correlated with changes in fiber volley amplitudes (simple linear regression analysis, comparison between slopes of lines) compared to their WT controls. Mean ± SEM. ns = *p* > 0.08.

**Figure S4.** NO-GC and GC-A expression in the auditory cortex of MR and GR^TMX^cKO mice. (A) MR^TMX^cKO mice showed significantly higher NO-GC and GC-A mRNA expression levels in comparison to MR WT mice. (B) GR^TMX^cKO mice showed significantly higher NO-GC mRNA expression levels and equal GC-A mRNA expression levels in comparison to GR WT mice. Mean. ns = p > 0.08, * = p < 0.05, ** = p < 0.01.

**Figure S5**. Location of the detected GRE binding motif within the Mus musculus *Gucy1a1* gene. Of note, this gene is located on the reverse strand and visualized as such. This figure is adapted from the view modes within Benchling.

**Figure S6**. Location of the detected GRE binding motif within the Mus musculus *Gucy1a2* gene. This figure is adapted from the view modes within Benchling.
