# Supplementary material for: Acute deletion of the central MR/GR steroid receptor correlates with changes in LTP, auditory neural gain, and GC-A cGMP signaling
Source: Front Mol Neurosci. 2023 Feb 17;16:1017761. doi: 10.3389/fnmol.2023.1017761 (PMC9983609; doi:10.3389/fnmol.2023.1017761)
Supplement: Supplementary file 2 [file Table_1.docx]

**Supplementary Table 1** Statistical information of the results

| Figure | Comparison | Statistical Test | Test Value | *p* - Value | Post-Hoc Test with *p* - Value | | *n* - Number |
| --- | --- | --- | --- | --- | --- | --- | --- |
| Fig. 1A | GR mRNA MR^TMX^cKO | two-tailed Student’s t-test | *t*(6) = 3.530 | *p* = 0.0124 |  | | MR WT: *n* = 4  MR cKO: *n* = 4 |
| Fig. 1B | MR mRNA GR^TMX^cKO | two-tailed Student’s t-test | *t*(8) = 1.993 | *p* = 0.0814 |  | | GR WT: *n* = 5  GR cKO: *n* = 5 |
| Fig. 2A |  |  |  |  | **Sidak’s multiple comparisons test** | |  |
|  | Wave IV/I Ratio MR^TMX^cKO | RM 2-way ANOVA | 2 genotypes  3 level ranges  F (1, 49) = 7.652 | *p =* 0.008 | MR WT vs MR^TMX^cKO | 10-30 dB: *p* = 0.4625 35-55 dB: *p* = 0.0378 60-80 dB: *p* = 0.0349 | MR WT: *n* = 30 ears of 16 mice  MR cKO: *n* = 21 ears of 14 mice |
|  | LTP MR^TMX^cKO | Mann-Whitney U test | U (117.8; 167.9) = 6 | *p* = 0.0093 |  | | MR WT: *n* = 7 ears of 4 mice  MR cKO: *n* = 8 ears of 5 mice |
| Fig. 2B |  |  |  |  | **Sidak’s multiple comparisons test** | |  |
|  | Wave IV/I Ratio GR^TMX^cKO | RM 2-way ANOVA | 2 genotypes  3 level ranges  F (1, 28) = 3.284 | *p =* 0.0807 | GR WT vs GR^TMX^cKO | 10-30 dB: *p* = 0.2579 35-55 dB: *p* = 0.4915 60-80 dB: *p* = 0.3631 | GR WT: *n* = 14 ears of 8 mice  GR cKO: *n* = 16 ears of 8 mice |
|  | LTP GR^TMX^cKO | Mann-Whitney U test | U (151.0, 124.0) = 12 | *p* = 0.0721 |  | | GR WT: *n* = 7ears of 5 mice  GR cKO: *n* = 8 ears of 6 mice |
| Fig. 2C | Wave IV/I Ratio MRGR^TMX^cKO | RM 2-way ANOVA | 2 genotypes  3 level ranges  F (1, 42) = 1.546 | *p* = 0.6962 | MRGR WT vs MRGR^TMX^cKO | 10-30 dB: *p* = 0.2579 35-55 dB: *p* = 0.4915 60-80 dB: *p* = 0.3631 | MRGR WT: *n* = 21 ears of 11 mice  MRGR cKO: *n* = 22 ears of 14 mice |
|  | LTP MRGR^TMX^cKO | Mann-Whitney U test | U (160.7, 121.3) = 45 | *p* = 0.0076 |  | | MRGR WT: *n* = 12 ears of 6 mice  MRGR cKO: *n* = 13 ears of 7 mice |
| Fig. 3A |  |  |  |  | **Sidak’s multiple comparisons test** | |  |
|  | PPF Ratio MR^TMX^cKO | 2-way ANOVA | F (1, 78) = 14.48 | *p* = 0.0003 | MR WT vs MR^TMX^cKO | *p* < 0.05 at 50 ms ISI | MR WT: *n* = 7 slices from 4 animals  MR cKO: *n* = 8 slices from 5 animals |
| Fig. 3B |  |  |  |  |  | |  |
|  | PPF Ratio GR^TMX^cKO | 2-way ANOVA | F (1, 78) = 2.172 | *p* = 0.1446 |  |  | GR WT: *n* = 7 slices from 5 animals  GR cKO: *n* = 9 slices from 6 animals |
| Fig. 3C | PPF Ratio MRGR^TMX^cKO | 2-way ANOVA | F (1, 138) = 2.217 | *p* = 0.1388 |  | | MRGR WT: *n* = 12 slices from 6 animals  MRGR cKO: *n* = 13 slices from 7 animals |
| Fig. 4A | NO-GC mRNA MR^TMX^cKO | two-tailed Student’s t-test | t(10) = 3.651 | *p* = 0.0045 |  | | MR WT: *n* = 6  MR cKO: *n* = 6 |
|  | Arc mRNA MR^TMX^cKO | two-tailed Student’s t-test | t(6) = 53.63 | *p* = 0.0001 |  | | MR WT: *n* = 4  MR cKO: *n* = 4 |
|  | GC-A mRNA MR^TMX^cKO | two-tailed Student’s t-test | t(8) = 6.874 | *p* = 0.0001 |  | | MR WT: *n* = 5  MR cKO: *n* = 5 |
| Fig. 4B | NO-GC mRNA GR^TMX^cKO | two-tailed Student’s t-test | t(8) = 3.924 | *p* = 0.0044 |  | | GR WT: *n* = 5  GR cKO: *n* = 5 |
|  | Arc mRNA GR^TMX^cKO | two-tailed Student’s t-test | t(6) = 0.4093 | *p* = 0.6965 |  | | GR WT: *n* = 4  GR cKO: *n* = 4 |
|  | GC-A mRNA GR^TMX^cKO | two-tailed Student’s t-test | t(8) = 0.3671 | *p* = 0.7231 |  | | GR WT: *n* = 5  GR cKO: *n* = 5 |
| Fig. 4C | NO-GC mRNA MRGR^TMX^cKO | two-tailed Student’s t-test | *t*(4) = 11.17 | *p* = 0.0004 |  | | MRGR WT: *n* = 3  MRGR cKO: *n* = 3 |
|  | Arc mRNA MRGR^TMX^cKO | two-tailed Student’s t-test | *t*(4) = 2.883 | *p* = 0.0449 |  | | MRGR WT: *n* = 3  MRGR cKO: *n* = 3 |
|  | GC-A mRNA MRGR^TMX^cKO | two-tailed Student’s t-test | *t*(4) = 5.941 | *p* = 0.0040 |  | | MRGR WT: *n* = 3  MRGR cKO: *n* = 3 |
|  |  |  |  |  |  | |  |
|  |  |  |  |  | **Sidak’s multiple comparisons test** | |  |
| Fig. S2A | Inter-Peak Latency MR^TMX^cKO | RM 2-way ANOVA | 2 genotypes  3 level ranges  F (1, 25) = 1.096 | *p* = 0.3050 | MR WT vs MR^TMX^cKO | 10-30 dB: *p* = 0.7710 35-55 dB: *p* = 0.7943 60-80 dB: *p* = 0.3713 | MR WT: *n* = 15 ears of 8 mice  MR cKO: *n* = 12 ears of 8 mice |
| Fig. S2B | Inter-Peak Latency GR^TMX^cKO | RM 2-way ANOVA | 2 genotypes  3 level ranges  F (1, 28 ) = 8.881 | *p* = 0.0059 | GR WT vs GR^TMX^cKO | 10-30 dB: *p* = 0.4540 35-55 dB: *p* = 0.092 60-80 dB: *p* = 0.004 | GR WT: *n* = 14 ears of 7 mice  GR cKO: *n* = 16 ears of 8 mice |
| Fig. S2C | Inter-Peak Latency MRGR^TMX^cKO | RM 2-way ANOVA | 2 genotypes  3 level ranges  F (1, 46) = 0.7076 | *p* = 0.4046 | MRGR WT vs MRGR^TMX^cKO | 10-30 dB: *p* = 0.9764 35-55 dB: *p* = 0.8671 60-80 dB: *p* = 0.4721 | MRGR WT: *n* = 21 ears of 11 mice  MRGR cKO: *n* = 27 ears of 14 mice |
|  |  |  |  |  |  | |  |
| Fig. S3A | fEPSP Slope MR^TMX^cKO | 2-way ANOVA | F (1, 72) = 1.218 | *p* = 0.2735 |  | | MR WT: *n* = 6 slices from 4 animals  MR cKO: *n* = 8 slices from 5 animals |
|  | FV Amplitude MR^TMX^cKO | 2-way ANOVA | F (1, 72) = 2.179 | *p* = 0.1443 |  | | MR WT: *n* = 6 slices from 4 animals  MR cKO: *n* = 8 slices from 5 animals |
|  | fEPSP Slope – FV Amplitude MR^TMX^cKO | Difference between regression lines (slopes) | F (1, 80) = 1.157 | *p* = 0.2854 |  | | MR WT: *n* = 6 slices from 4 animals  MR cKO: *n* = 8 slices from 5 animals |
| Fig. S3B | fEPSP Slope GR^TMX^cKO | 2-way ANOVA | F (1, 78) = 1.189 | *p* = 0.2788 |  | | GR WT: *n* = 7 slices from 4 animals  GR cKO: *n* = 7 slices from 5 animals |
|  | FV Amplitude GR^TMX^cKO | 2-way ANOVA | F (1, 78) = 1.203 | *p* = 0.2761 |  | | GR WT: *n* = 7 slices from 4 animals  GR cKO: *n* = 7 slices from 5 animals |
|  | fEPSP Slope – FV Amplitude GR^TMX^cKO | Difference between regression lines (slopes) | F (1, 86) = 2.156 | *p* = 0.1456 |  | | GR WT: *n* = 7 slices from 4 animals  GR cKO: *n* = 7 slices from 5 animals |
| Fig. S3C | fEPSP Slope MRGR^TMX^cKO | 2-way ANOVA | F (1, 126) = 0.0237 | *p* = 0.8780 |  | | MRGR WT: *n* = 11 slices from 6 animals  MRGR cKO: *n* = 11 slices from 7 animals |
|  | FV Amplitude MRGR^TMX^cKO | 2-way ANOVA | F (1, 126) = 0.0059 | *p* = 0.9390 |  | | MRGR WT: *n* = 11 slices from 6 animals  MRGR cKO: *n* = 13 slices from 7 animals |
|  | fEPSP Slope – FV Amplitude MRGR^TMX^cKO | Difference between regression lines (slopes) | F (1, 134) = 1.074 | *p* = 0.3019 |  | | MRGR WT: *n* = 11 slices from 6 animals  MRGR cKO: *n* = 13 slices from 7 animals |
|  |  |  |  |  |  | |  |
| Fig. S4A | NO-GC mRNA MR^TMX^cKO | two-tailed Student’s t-test | *t*(6) = 2.520 | *p* = 0.0453 |  | | MR WT: *n* = 4  MR cKO: *n* = 4 |
|  | GC-A mRNA MR^TMX^cKO | two-tailed Student’s t-test | *t*(4) = 4.989 | *p* = 0.0075 |  | | MR WT: *n* = 3  MR cKO: *n* = 3 |
| Fig. S4B | NO-GC mRNA GR^TMX^cKO | two-tailed Student’s t-test | *t*(6) = 4.834 | *p* = 0.0029 |  | | GR WT: *n* = 4  GR cKO: *n* = 4 |
|  | GC-A mRNA GR^TMX^cKO | two-tailed Student’s t-test | *t*(6) = 0.4926 | *p* = 0.6398 |  | | GR WT: *n* = 4  GR cKO: *n* = 4 |
|  |  |  |  |  |  | |  |
