# Supplementary figures and images for: Acute deletion of the central MR/GR steroid receptor correlates with changes in LTP, auditory neural gain, and GC-A cGMP signaling
Source: Front Mol Neurosci. 2023 Feb 17;16:1017761. doi: 10.3389/fnmol.2023.1017761 (PMC9983609; doi:10.3389/fnmol.2023.1017761)

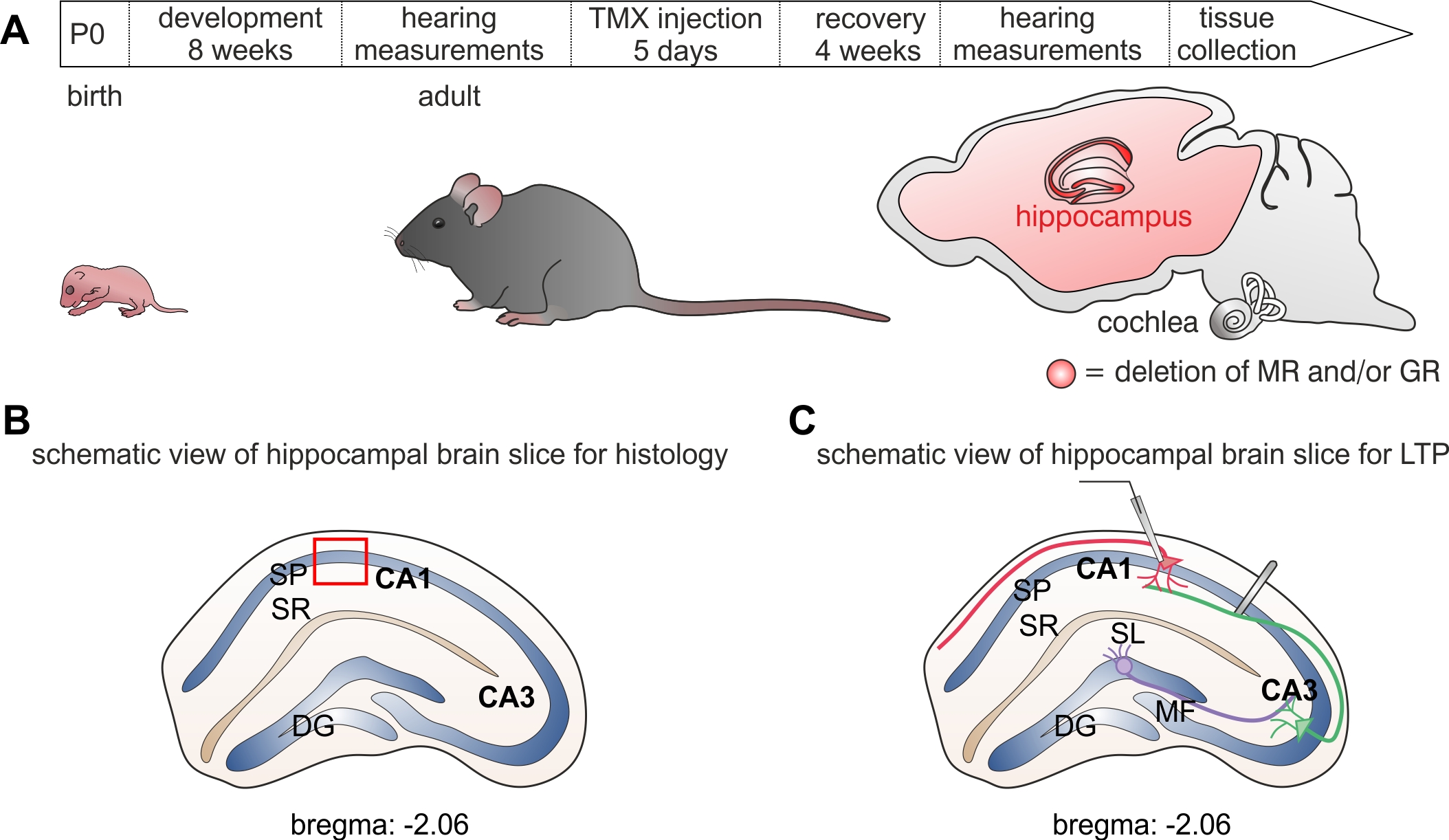

Supplement: Supplementary file 3 [file Image_1.JPEG]

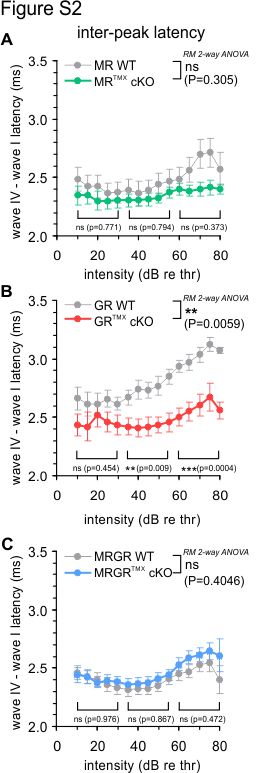

Supplement: Supplementary file 4 [file Image_2.JPEG]

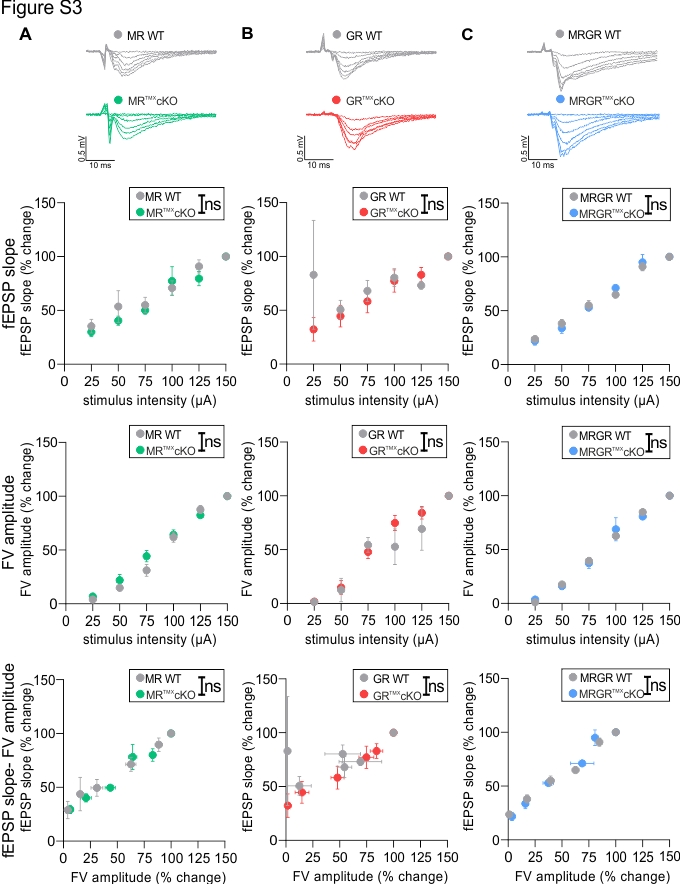

Supplement: Supplementary file 5 [file Image_3.JPEG]

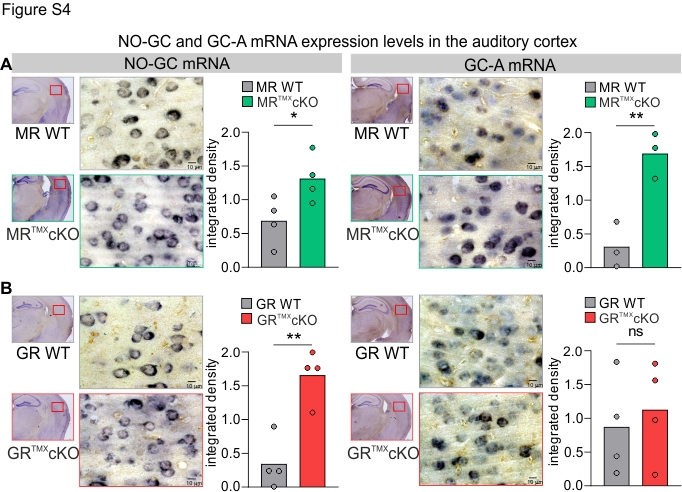

Supplement: Supplementary file 6 [file Image_4.JPEG]

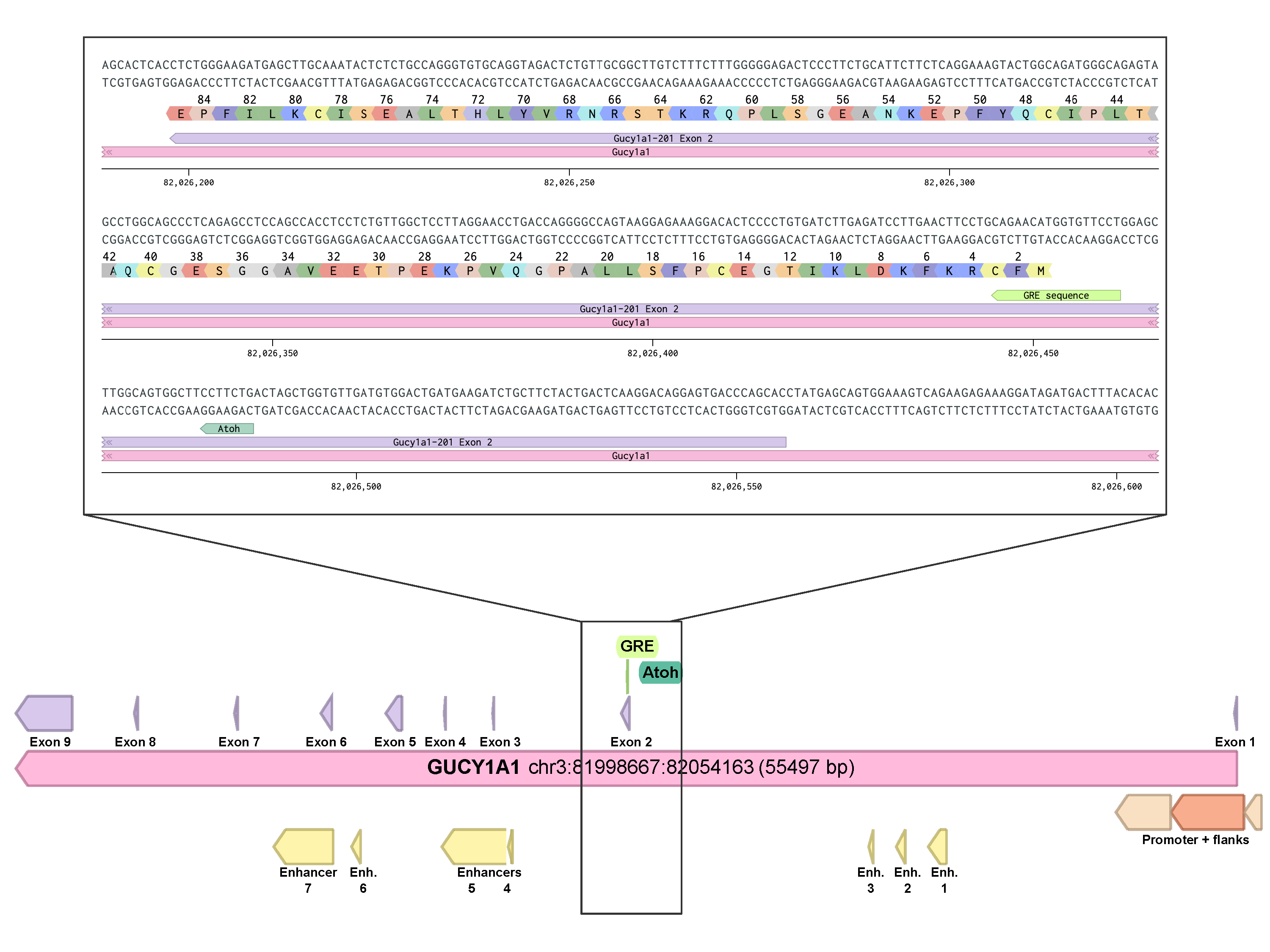

Supplement: Supplementary file 7 [file Image_5.TIFF]

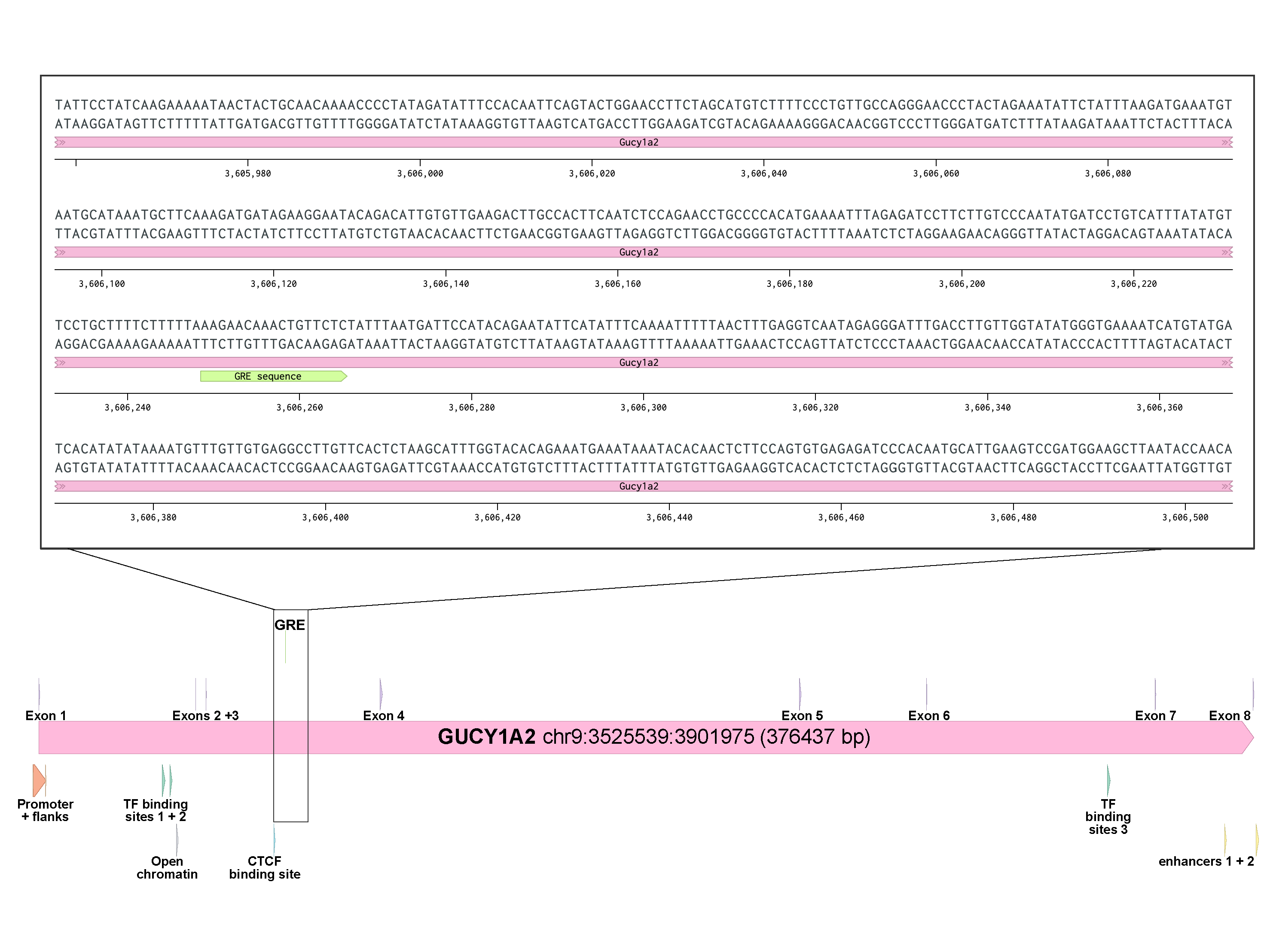

Supplement: Supplementary file 8 [file Image_6.TIF]
